# Preparation and Application of Macromolecular Silane Coupling Agent for Polyimide-Based Composites

**DOI:** 10.3390/ma19163435

**Published:** 2026-08-13

**Authors:** Jianquan Li, Xiang Li, Ziyong Liang, Huailin Fan, Qingyu Ma

**Affiliations:** School of Materials Science and Engineering, University of Jinan, Jinan 250022, China; mse_lijq@ujn.edu.cn (J.L.); huailinfan@163.com (H.F.)

**Keywords:** polyimides, macromolecular silane coupling agents, composite materials

## Abstract

This study presents targeted contributions to the development of macromolecular silane coupling agents (MSCAs) and high-performance fiber-reinforced polyimide (PI) composites. Three novel MSCAs were synthesized via chemical imidation and transamidation reactions, using hexafluoroisopropylidene diphthalic anhydride and 2,3,3′,4′-diphenyl ether tetracarboxylic acid as dianhydride monomers, 4,4′-diaminodiphenyl ether and 1,3-bis(4′-aminophenoxy)benzene as diamine monomers, and aminopropyltriethoxysilane (KH550) as the capping agent. Structural characterization by FTIR, ^1^H NMR, and XPS confirmed the successful synthesis of the target products, with silicon contents of 3.29%, 3.37%, and 3.66%, respectively. The MSCAs exhibited excellent thermal stability, with 10% weight loss temperatures ranging from 462 °C to 543.3 °C, and good solubility in most polar organic solvents, addressing the poor processability of conventional macromolecular coupling agents. Compared with small-molecule KH550, the MSCAs significantly enhanced interfacial properties: the average tensile and flexural strengths of the composites increased by 11.0% and 9.8%, respectively, compared to 3.9% and 4.0% for KH550. SEM analysis demonstrated that MSCAs improved resin adhesion to fibers and fiber–resin compatibility. Additionally, the T5, T10, and glass transition temperatures of the composites were further optimized due to polymer chain diffusion and entanglement. This work provides a feasible strategy for interfacial design in high-performance polyimide composites.

## 1. Introduction

Polyimide (PI) is an important polymer material with excellent comprehensive properties, which is widely used in aerospace, military, home appliances, microelectronics, the chemical industry, energy conservation and environmental protection, and other fields [1]. Its main chain contains imide group (-R-CO-NH-CO-R’-), and PI is prepared via a multi-step condensation reaction of dianhydrides and diamines. PI exhibits outstanding heat resistance [2], low temperature resistance [3], corrosion resistance [4], radiation resistance, aging resistance, good electrical insulation, excellent mechanical properties, and low dielectric loss, making it known as one of the most promising engineering plastics in the 21st century [5].

Silane coupling agents, developed in the 1940s for glass-fiber-reinforced plastics [6], have become indispensable additives in composites, organosilicon, and other fields due to their dual-functional structure (carbon groups reacting with organics, silicon groups bonding with inorganics) [7,8], with expanding application scopes.

Divided into small-molecule (e.g., KH550, KH570) and macromolecular types, their performance differs significantly. Small-molecule ones, with simple synthesis and low cost [9], have inherent defects: short chains cause thin coating layers, and single active groups limit interface interaction, leading to poor long-term compatibility [10].

In contrast, macromolecular silane coupling agents, with long chains and multiple active groups, have better hydrophobicity and interface binding ability. They form multi-point bonding with polymers and inorganics, improving composite stability [11]. Small-molecule types can no longer meet high-end industrial demands [12], making the development of targeted macromolecular ones a research focus.

Sekhavat Pour et al. 2018 synthesized a macromolecular coupling agent (MCA) containing epoxides and silanol functional groups via copolymerization, and successfully grafted it onto the surface of TiO_2_ nanoparticles [13]. The TiO_2_ nanoparticles were mixed with epoxy resin and coated on low carbon steel. It was found that the uniform mixing and interface bonding of MCA exerted a good effect on the long-term corrosion resistance of the steel, and improved the mechanical properties of TiO_2_ nanocomposites. Yan et al. 2014 developed a new macromolecular silane coupling agent, which was prepared by mixing 2,2′-Bis(4-cyanophenyl) isopropyl methylbiphenyl with modified nano-SiO_2_ [14]. The results show that the modified SiO_2_ particles are uniformly dispersed in the matrix, which significantly improves the impact strength, bending strength, and glass storage modulus of the composites, and also increases the glass transition temperature. These performance improvements are attributed to changes in interface adhesion and dispersity of the filler. Crespy et al. 1992 synthesized a macromolecular coupling agent by the reaction between a polymer with an end group (prepared by anionic polymerization) and an alkoxysilane or epoxy group; this coupling agent is used in glass-fiber-reinforced composites, acting as a bonding and coupling agent [15]. Cheng et al. 2014 modified titanium nitride (TiN) nanoparticles by the direct mixing method using a randomly copolymerized functionalized macromolecular coupling agent (F-MCA) [16]. The results showed that F-MCA formed an organic coating layer and covalent bonds with the surface of TiN particles. The modified TiN particles had good dispersion in ethyl acetate and increased surface hydrophobicity. These modified TiN particles can be used in various polymer mixtures and nanocomposites to improve performance and service life.

At present, the coupling agents used for polyimide-based composite materials are mainly small-molecule silane coupling agents. Although modifying the surface of the reinforcing material can achieve a certain reinforcement effect, due to compatibility issues, the interaction efficiency between the fibers and the matrix resin is low, so the reinforcing effect is also very limited. Compared with small-molecule coupling agents, macromolecule coupling agents have relatively long molecular chains [17]. When used to modify polyimide-based composite materials, they can form stronger physical entanglement with the polymer matrix, achieving effective reinforcement; moreover, the structure and composition of the synthesized macromolecular coupling agents can be adjusted through design to control and improve the performance of the materials [18,19].

Starting from the actual demand and future development of polyimide composite, this article aims to solve the problems of weak interaction between small-molecule silane coupling agents and PI resin, as well as the unsatisfactory reinforcement effect of composite materials. Based on molecular design, a macromolecular silane coupling agent with a polyimide polymer skeleton and flexible winding characteristics was synthesized; this coupling agent was used to modify the surface of glass fibers and carbon fibers for the preparation of polyimide-based composite materials. By controlling the structure of macromolecular coupling agents, the physical, mechanical, and processing properties of the composite materials can be improved, thereby obtaining high-performance fiber/polyimide composites.

## 2. Materials and Methods

### 2.1. Materials and Chemicals

Hexafluoroisopropylidene diphthalic anhydride (6FDA), diaminodiphenyl ether (ODA), *N*,*N*-dimethylacetamide (DMAc), 2-aminopyridine (2APY), acetic anhydride, triethylamine, 2,3,3′,4′-diphenyl ether tetracarboxylic anhydride (a-ODPA), 1,3-Bis(4′-aminophenoxy)benzene (1,3,4-APB), 3-aminopropyltriethoxysilane (KH550), anhydrous ethanol.

### 2.2. Preparation of Polyimide-Based Macromolecular Silane Coupling Agent

**Figure 1:**
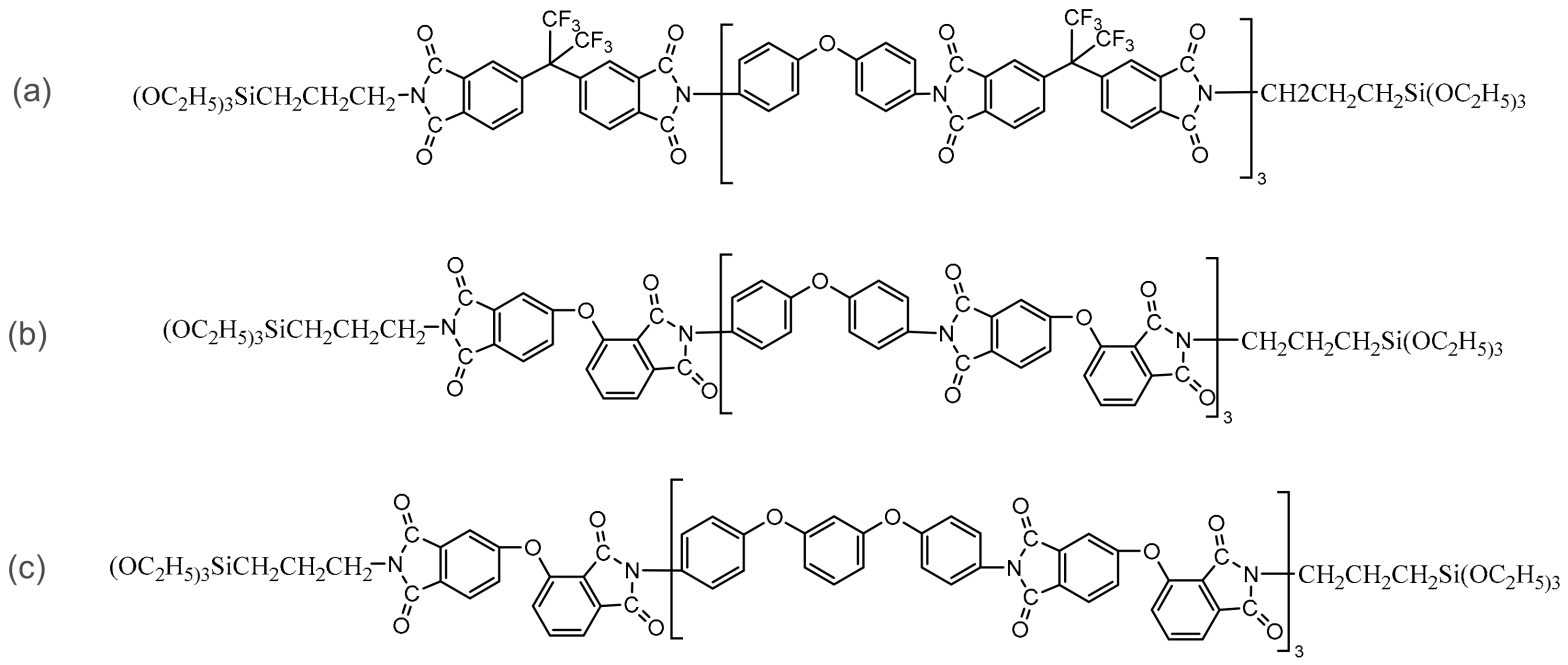
(**a**) 6FDA-ODA-KH550 structural formula. (**b**) A-ODPA-ODA-KH550 structural formula. (**c**) A-ODPA-1,3,4APB-KH550 structural formula.

This article designs three types of macromolecular silane coupling agents. Firstly, a 2-aminopyridine terminated polyamide acid oligomer was synthesized using a feed ratio of dianhydride: diamine: diaminopyridine = 4:3:2. The oligomer was then cyclodehydrated via chemical imidization. Subsequently, the final product was synthesized through a transfer acylation reaction. Figure 1 shows the structural formulas of three designed macromolecular silane coupling agents.

The experimental procedures, raw materials, and yields for the preparation of the three macromolecular silane coupling agents are listed in Table 1.

**Figure 2:**
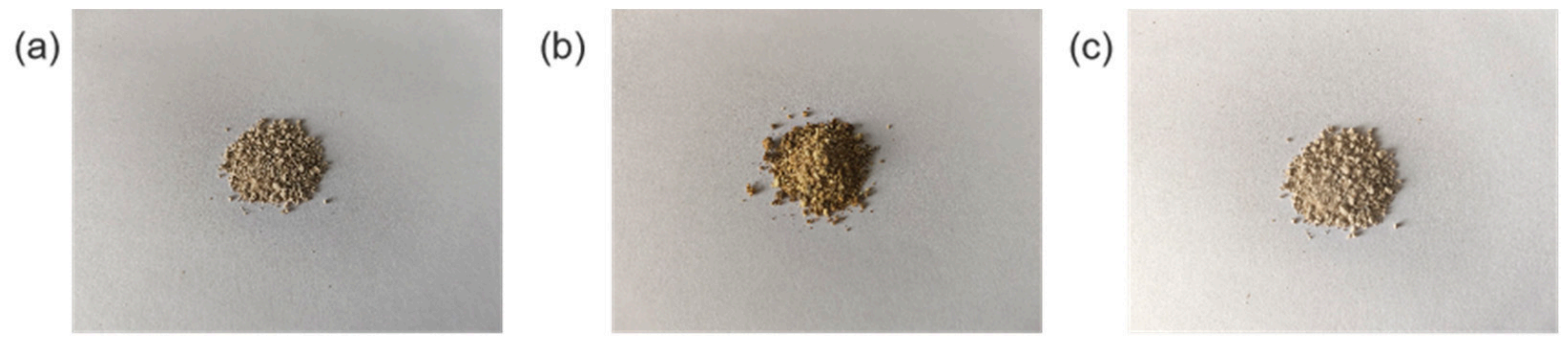
(**a**) 6FDA-ODA-KH550 powder. (**b**) a-ODPA-ODA-KH550 powder. (**c**) a-ODPA-1,3,4APB-KH550 powder.

Three types of macromolecular silane coupling agent powders: 6FDA-ODA-KH550, a-ODPA-ODA-KH550, and a-ODPA-1,3,4APB-KH550, as shown in Figure 2a–c.

### 2.3. Effect of Macromolecular Silane Coupling Agent on Polyimide Matrix Composites

#### 2.3.1. Preparation of Silane Coupling Agent Modified Fiber

In this experiment, the average length of the cut glass fiber was 3 mm and the glass fiber powder had a mesh size of 325. Before the surface modification of the glass fibers, they were first cleaned by soaking in an acetone solution for 24 h, and subjecting to ultrasonic treatment for 30 min. Subsequently, the glass fibers were washed with distilled water to remove the acetone solution and impurities, and then dried in a vacuum oven for 24 h. Si−OH groups are formed by hydrolysis of the silane coupling agent, which can couple with the Si−OH groups on the glass fiber surface under certain conditions. The three kinds of large macromolecule silane coupling agents prepared in this study and a small-molecule silane coupling agent (KH550) were used for the surface coupling of the glass fiber. Firstly, a 2% ethanol solution of KH550 and a DMAc solution of macromolecular silane coupling agent were prepared with a ratio of silane coupling agent: solvent: water ratio of 5:9 4:1, followed by magnetic stirring for 10 min. The cleaned and dried glass fibers were then placed into the hydrolysate of silane coupling agent, subjected to ultrasonic treatment for 10 min, and the oil bath temperature was set at 80 °C, for a reaction duration of 12 h [20]. The treated glass fibers were filtered into a beaker, and the unreacted coupling agent on the surface was removed by ultrasonic cleaning. Finally, the glass fibers were dried in a drying oven at 80 °C for 24 h.

The average length of the carbon fibers was 3 mm, and the carbon fiber powder had a mesh size of 325. Due to the low surface activity of carbon fiber, it is difficult to graft silane coupling agent onto their surfaces [21]. In this study, nitric acid was used to oxidize the surface of carbon fiber to generate oxygen containing active groups such as the hydroxyl group, thereby improving their surface activity [22]. Firstly, the surface of carbon fibers was cleaned by soaking them in anhydrous ethanol solution for 24 h, followed by ultrasonic treatment for 30 min, and vacuum drying. The dried carbon fibers were treated with nitric acid in a water bath at 60 °C. After treatment, the carbon fibers were washed with distilled water until the pH was close to 7, and then dried in a vacuum drying oven. The carbon fibers were then immersed in the hydrolysate of the configured silane coupling agent and ultrasonically dispersed for 10 min. After the oil bath was heated to 80 °C and subjected to condensation reflux for 12 h, the unreacted silane coupling agent on the surface was removed by suction filtration and cleaning. Finally, the carbon fibers were placed in a vacuum drying oven and dried at a constant temperature of 80 °C.

#### 2.3.2. Preparation of Polyimide Matrix Composites

First, the pure PI resin was dried in a constant temperature drying oven at 20 °C for more than 12 h. Subsequently, fiber powder with a mass fraction of 10% was mixed with the polyimide matrix. Uniform mixing of the fiber powder and PI-2542 powder was achieved via physical stirring. Meanwhile, static electricity generated by mutual friction between particles during stirring enabled the fiber powder to adhere evenly to the surface of the matrix particles. The extruder and granulator were switched on, and the temperature of each barrel zone was preset in advance. After continuous preheating, the actual temperature of each barrel section reached 365 °C, 365 °C, 360 °C, 360 °C, 360 °C, 360 °C, 360 °C, and 370 °C, respectively. Specifically, the temperature of the main feeding port was maintained at 365 °C, the temperature of the middle barrel zone was set to 360 °C, and the temperature of the extrusion outlet was controlled at 370 °C. After the barrel temperature remained stable for a certain period, the extrusion and granulation experiment could be formally carried out. The homogeneous mixture of PI matrix and fiber powder was quantitatively fed into the extruder through the main feeding port, and timing was initiated simultaneously. Under high-temperature shear environment inside the barrel, the PI matrix was fully melted, and the predispersed fiber powder was uniformly distributed in the molten resin. The blended melt was continuously conveyed to the extrusion die outlet by the rotating screw. When stable melt extrusion began at the die outlet, the initial residence time t_1_ was recorded as 45 s. Afterwards, the timer was reset for subsequent continuous recording. The extruded fiber-reinforced PI composite strands were collected, and a small segment of unstable frontend strands was discarded to ensure uniform material quality. Then, the rotating speed of the granulator was adjusted to a matched rate for uniform pelletizing, and the fiber-powder-reinforced PI composite pellets were finally obtained. The feeding operation was stopped when no melt was extruded from the die outlet, and the total continuous extrusion time t_2_ was recorded as 2 min and 50 s. Notably, the pretreated 3 mm shortcut staple fibers presented a fluffy loose state, which was not suitable for direct feeding through the main feeding port to avoid agglomeration and uneven feeding. Therefore, a side forced feeding mode was adopted for staple fiber addition. In the formal experiment, the pure PI matrix was first added through the main feeding port. After reaching the preset residence time t_1_, 3 mm shortcut staple fibers with a mass fraction of 10% were continuously added through the side forced feeding port, and the entire fiber feeding process was completed within the recorded time t_2_. Meanwhile, the continuous extruded composite strands from the die outlet were immediately cooled and shaped by a water-cooled drafting device to obtain uniform 3 mm short-cut-fiber-reinforced PI composite strands. Similarly, the unstable frontend part of the composite strands was removed, and the granulator speed was reasonably adjusted for precision pelletizing. Finally, the standard 3 mm short-cut-fiber-reinforced PI composite granular materials were prepared for subsequent performance testing.

**Figure 3:**
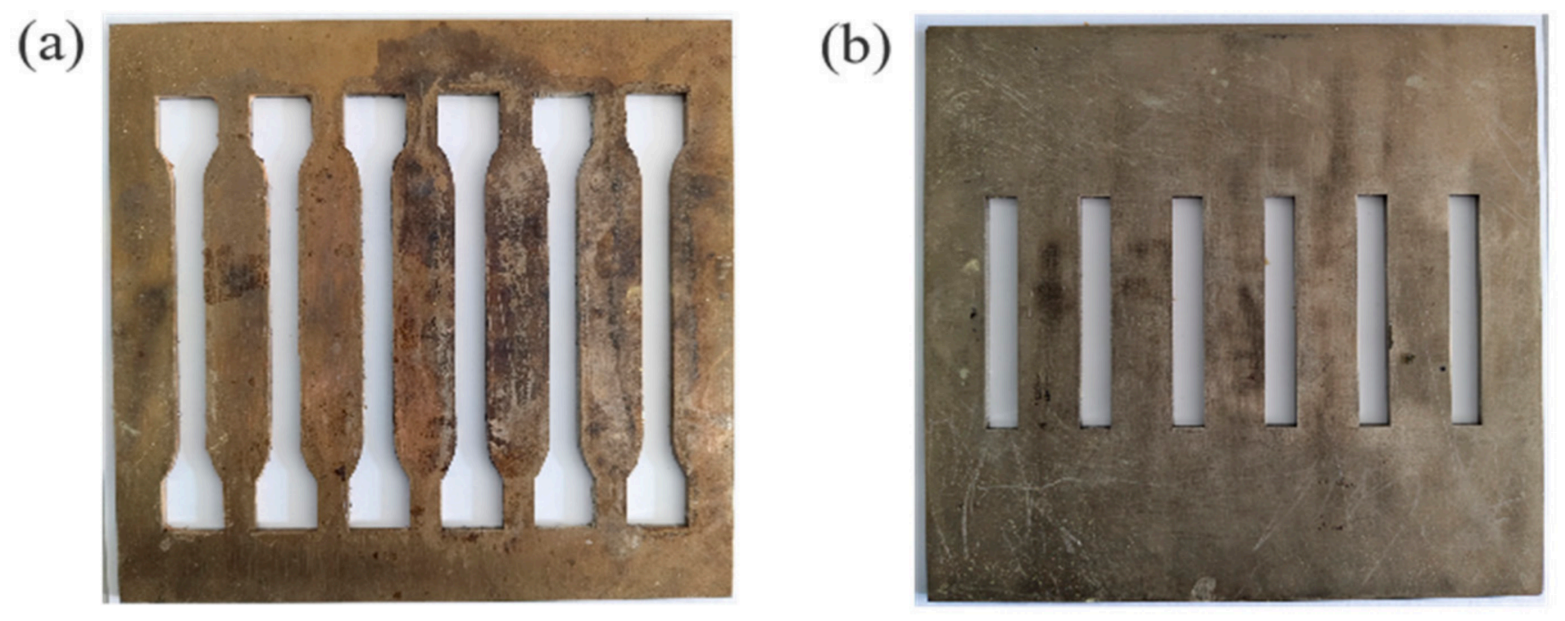
(**a**) Stretch spline die. (**b**) Curved spline die.

The prepared granules were placed in a drying oven and dried at 150 °C for 4 h to remove moisture. An automatic hot press was used for hot molding. A dumbbell-shaped tensile spline (length: 150 mm wide: 20 mm, 10 mm thick: 4 mm) and a long curved spline (length: 80 mm wide: 10 mm thick: 4 mm) were made, respectively. The mold is 40 cm × 40 cm, as shown in Figure 3a,b. In order to facilitate demolding, a layer of release agent was sprayed on the surface of the mold, and 12 g of granules was added into the tensile spline groove of the mold, and 5 g of granules was added to the bending spline groove. The parameters of the automatic hot press were set as follows: temperature of 365 °C, holding time of 30 min, upper pressure limit of 25.0 T, and lower pressure limit of 24.7 T, after which heating was started. When the temperature rose to 250 °C, the mold containing the granules to be molded was placed between the two heating devices, and a prepressure of 2–4 T was applied. When the temperature reached 360 °C, pressure was applied until the preset pressure of 25 T was achieved. After holding the pressure for 30 min, the temperature was allowed to cool naturally. When the temperature dropped to 150 °C, the pressure could be released for demudding.

## 3. Results and Discussion

This article uses hexafluoroisopropylidene diphthalic anhydride, 4,4′-diaminodi-phenyl ether, 2,3,3′,4′-diphenyl ether tetracarboxylic anhydride, and 1,3-bis (4′-ami-nophenoxy) as raw materials to prepare three types of macromolecular silane coupling agents via the chemical imidization method. During the imidization process, polyamide acid needs to undergo dehydration and cyclization, while alkoxysilane tends to hydroly ze in the presence of water to form silanols. Therefore, in the synthesis of macromolecular silane coupling agents, 2-aminopyridine is first used to terminate the oligomer of polyimide, and then a silane coupling agent is introduced through transacylation to prevent the terminated silane coupling agent from hydrolyzing prematurely during the dehydration cyclization step. The structures were characterized by Fourier transform infrared spectroscopy (FT-IR), nuclear magnetic resonance hydrogen spectroscopy (^1^H NMR), X-ray photoelectron spectroscopy (XPS), and other methods. The physicochemical properties were analyzed by gel permeation chromatography (GPC), thermogravimetry (TG), solubility tests, and other techniques.

### 3.1. Structural Characterization

#### 3.1.1. Analysis of Fourier Transform Infrared Spectroscopy

**Figure 4:**
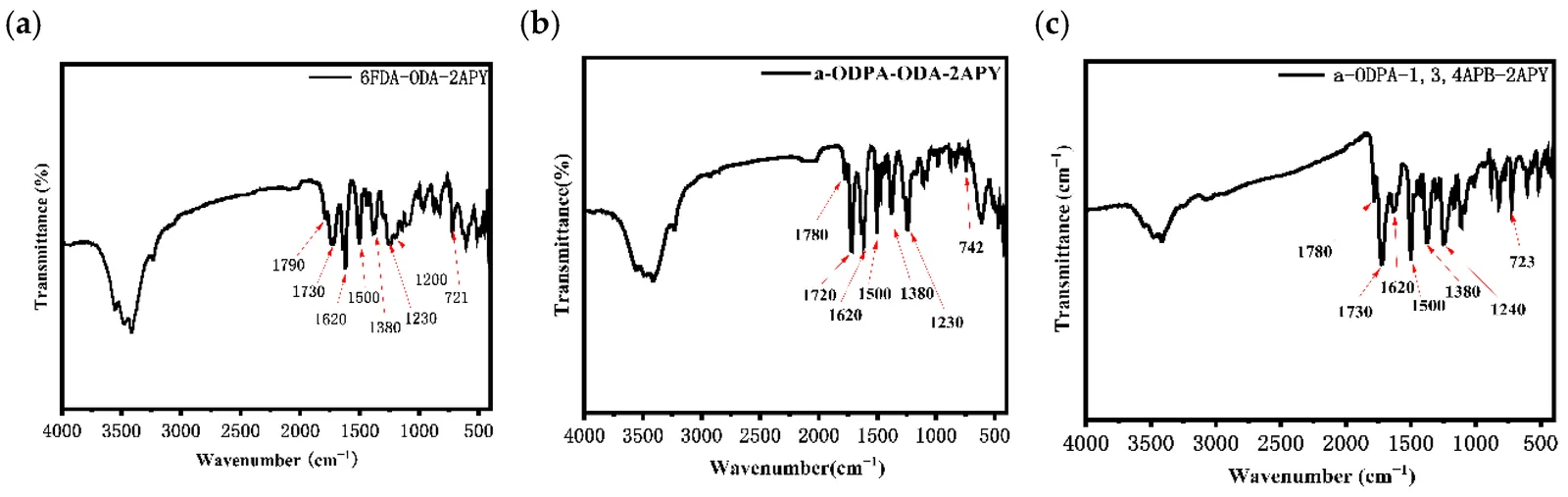
(**a**) FT-IR of 6FDA-ODA-2APY. (**b**) FT-IR of a-ODPA-ODA-2APY. (**c**) FT-IR of a-ODPA-1,3,4APB-2APY.

Figure 4a–c present the FT-IR spectra of the three 2-aminopyridine-terminated polyimide oligomers, and distinct structural differences can be observed among the samples. All three products exhibit characteristic absorption peaks of typical polyimide structures [23], including the asymmetric and symmetric C=O stretching vibrations of the imide ring at approximately 1780 cm^−1^ and 1730 cm^−1^, the imide ring deformation peak at 743 cm^−1^, the C–N stretching peak at 1380 cm^−1^, and the benzene ring vibration peak at 1500 cm^−1^. The absence of the CONH carbonyl peak near 1650 cm^−1^ confirms that thermal imidization was sufficiently complete in all samples.

Notable differences exist among the three oligomers. 6FDA-ODA-2APY (Figure 4a) shows an additional strong absorption peak at 1200 cm^−1^ corresponding to C–F stretching vibrations derived from the 6FDA monomer, which is absent in the a-ODPA-based samples.

In contrast, a-ODPA-ODA-2APY and a-ODPA-1,3,4APB-2APY display stronger ether bond (C–O–C) absorption intensities near 1230 cm^−1^ due to the presence of ether linkages in a-ODPA and the diamine monomers. Furthermore, the spectrum of a-ODPA-1,3,4APB-2APY shows relatively higher peak intensity at 1500 cm^−1^ associated with benzene ring vibrations, owing to the more aromatic structure of 1,3,4APB.

Overall, although all three polyimide oligomers show complete imidization and similar characteristic imide peaks, their FT-IR spectra clearly reflect structural differences introduced by distinct dianhydride and diamine monomers. These differences confirm the successful preparation of the designed polyimide precursors and lay the foundation for subsequent synthesis of macromolecular silane coupling agents.

**Figure 5:**
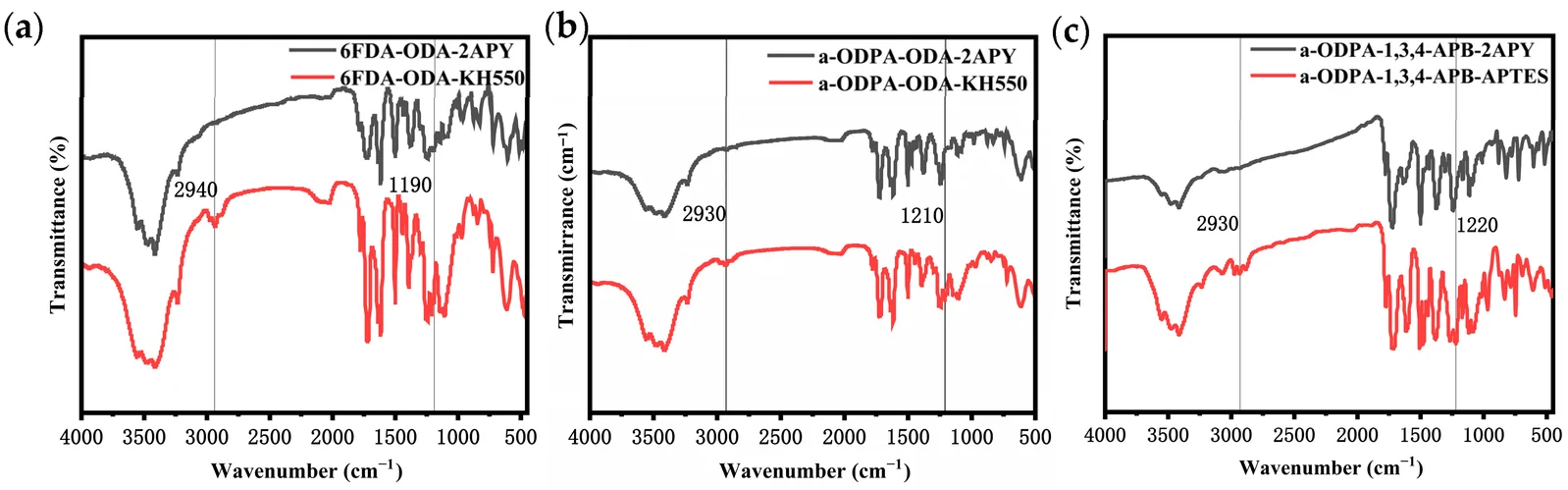
(**a**) FTIR of 6FDA-ODA-2APY and 6FDA-ODA-KH550. (**b**) FTIR of a-ODPA-ODA-2APY and a-ODPA-ODA-KH550. (**c**) FTIR of A-ODPA-1,3,4APB-2APY and A-ODPA-1,3,4APB-KH550.

Figure 5a–c show the FT-IR spectra of polyimide oligomers terminated with 2-aminopyridine and KH550. After comparison, an additional infrared absorption peak appears at around 1210 cm^−1^ in the KH550-terminated polyimide oligomers, which corresponds to the stretching vibration of the Si-O bond [24]. This indicates that the 2-aminopyridine-terminated groups undergo a transfer acylation reaction with KH550, introducing the Si-O bond. At 2930 cm^−1^, there is a stretching vibration peak of methylene, which is attributed to the aminopropyl group on KH550 [25], further indicating that 2-aminopyridine has been successfully replaced by KH550.

#### 3.1.2. NMR

**Figure 6:**
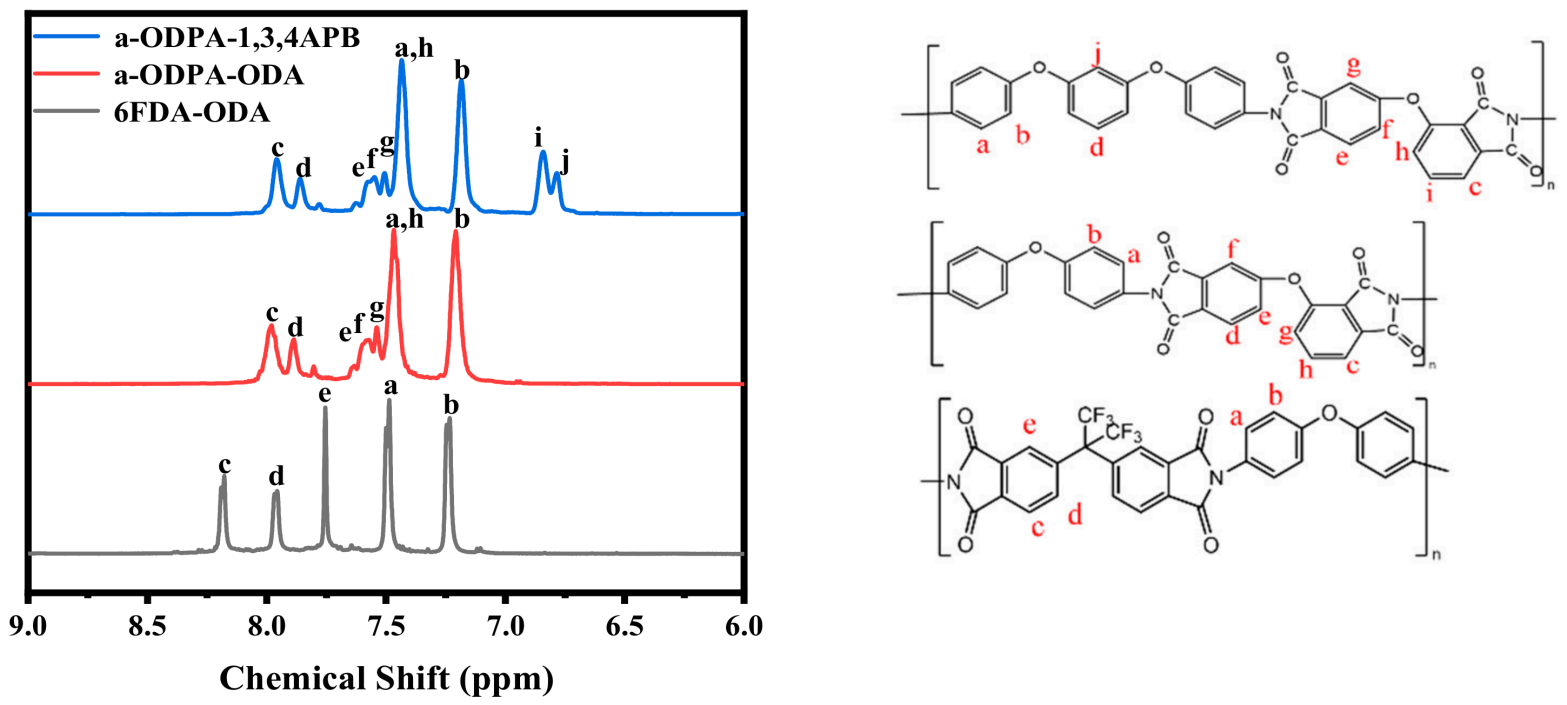
NMR hydrogen spectra of polyimide oligomers. In the blue spectrum, peaks a and b are assigned to the protons on the terminal benzene rings of the diamine (APB) moiety, while peaks d and j correspond to the protons on its central meta-substituted benzene ring. Furthermore, peaks c and e–i represent the protons on the benzene rings of the asymmetric dianhydride (a-ODPA) moiety. In the red spectrum, peaks a and b denote the protons on the benzene rings of the diamine (ODA) moiety. Because a-ODPA is an asymmetric dianhydride with protons in different chemical environments across its two benzene rings, six distinct letters (c–h) are required to respectively label these unique proton positions. Finally, in the black spectrum, peaks a and b correspond to the protons on the benzene rings of the diamine (ODA) moiety, whereas peaks c, d, and e represent the protons at three different positions on the benzene rings of the dianhydride (6FDA) moiety that appear in the higher chemical shift region.

The ^1^H NMR spectra clearly demonstrate the molecular structural characteristics of the three 2-aminopyridine-end-capped polyimide oligomers (a-ODPA-1,3,4APB, a-ODPA-ODA, 6FDA-ODA). Peak integration, attribution characterization, and end group analysis triple-verification confirm the successful synthesis (Figure 6). 

Verification of Molecular Structure and Peak Integration: All samples exhibit a polyimide main chain aromatic hydrogen signal spanning 7.0–8.2 ppm. Samples containing ODA (6FDA-ODA, a-ODPA-ODA) display characteristic peaks at ~7.2 ppm and ~7.5 ppm, with an integration ratio of 1:1, corresponding to ODA benzene ring hydrogens (4:4), confirming the successful incorporation of ODA. For a-ODPA-based samples, the overlapping peak integrals e, f, g in the 7.5–8.1 ppm range are consistent with the theoretical number of hydrogens specified for a-ODPA, verifying the accurate feeding of the dianhydride. Similarly, the i, j peaks corresponding to 1,3,4APB aromatic hydrogens in a-ODPA-1,3,4APB at 6.5–7.0 ppm show integration ratios matching the structural theory. No CONH active hydrogen signal is observed at ~10 ppm throughout the spectra, fully proving the completion of imidization.

End-Group Analysis: All three samples exhibit characteristic peaks of the 2APY end group at 6.5–7.0 ppm, with integration areas matching the design value of “2 end groups per oligomer molecule,” confirming the completion of the end capping reaction and providing sites for subsequent silane grafting.

In summary, the peak patterns, integration ratios, and end-group signals are highly consistent with the molecular design, strongly confirming the successful preparation of the three oligomers.

**Figure 7:**
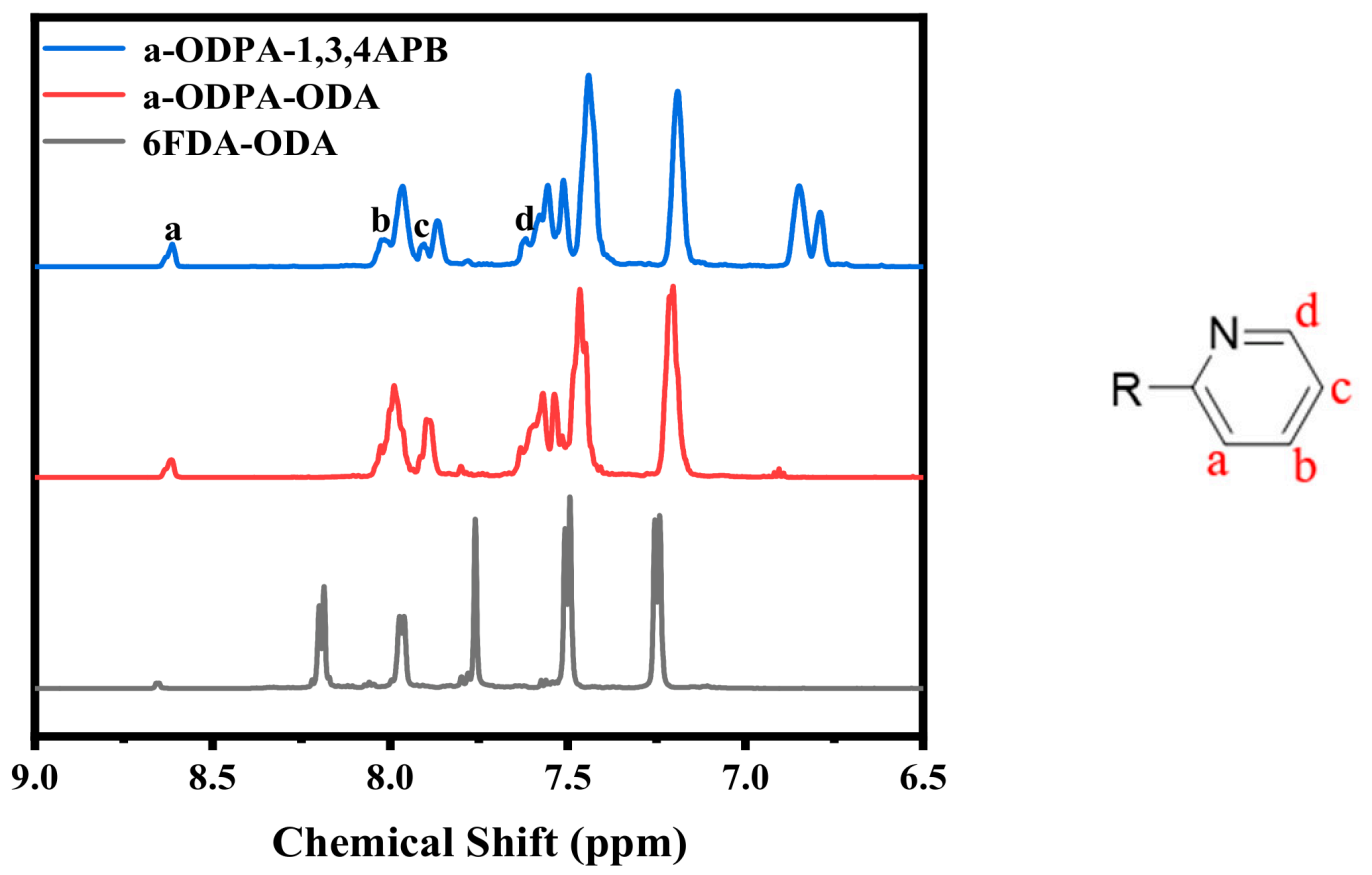
NMR hydrogen spectra of 2-aminopyridine terminated polyimide oligomers. Peak a is assigned to the proton directly adjacent to the nitrogen atom on the pyridine ring while peaks b–d correspond to the other three protons on the pyridine ring.

Figure 7 shows the nuclear magnetic resonance hydrogen spectrum (^1^H NMR) of 2-aminopyridine-terminated polyimide oligomers. Hydrogen a corresponds to 8.61 ppm, hydrogen b to 8.06 ppm, hydrogen to 7.79 ppm, and hydrogen d to 7.67 ppm; all of these are peaks of the pyridine ring after termination [26]. This result proves that 2-aminopyridine has successfully terminated at both ends of the polyimide chains.

**Figure 8:**
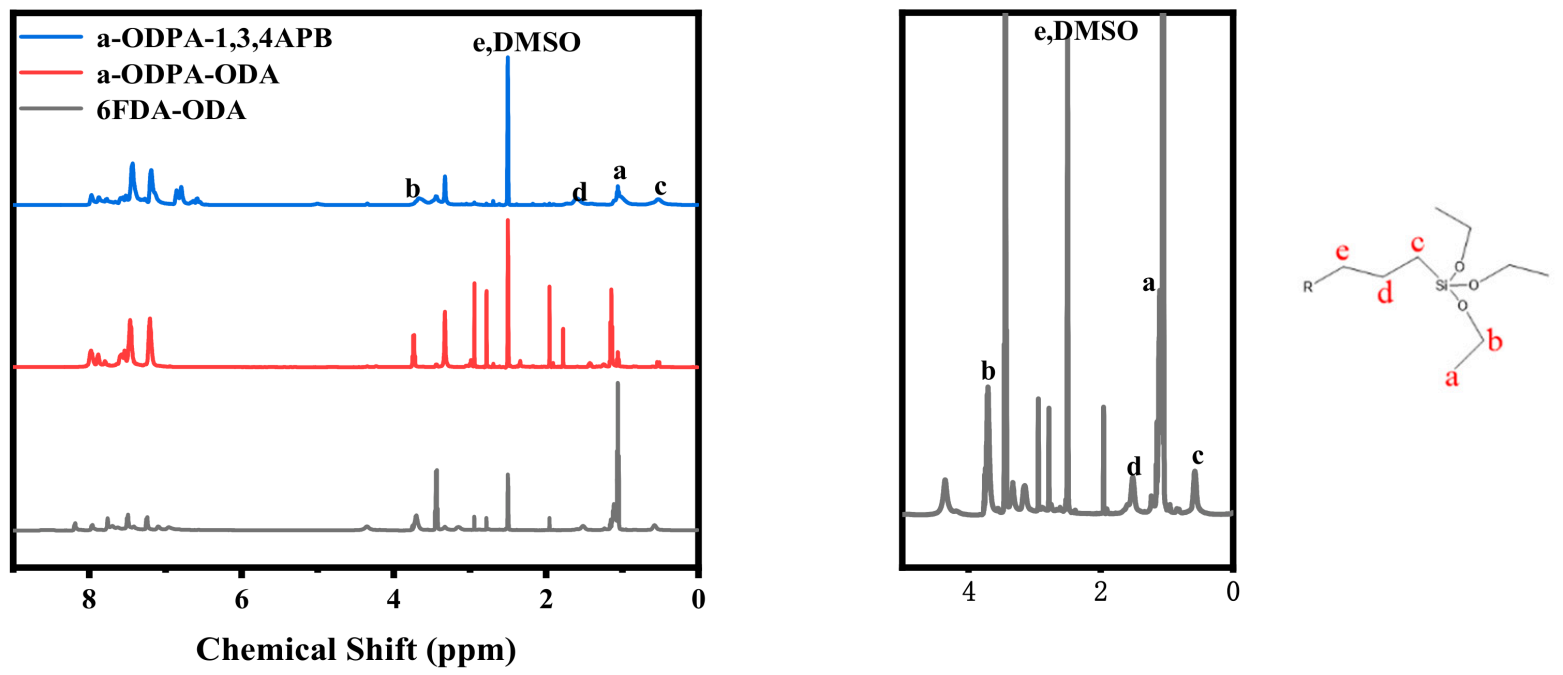
NMR hydrogen spectra of three macromolecular silane coupling agents. In the ^1^H NMR spectrum, peak a is assigned to the methyl protons of the ethoxy groups while peak b corresponds to the methylene protons directly bonded to the oxygen atom within the ethoxy moiety. Peak c represents the methylene protons directly attached to the silicon atom and peak d is attributed to the methylene protons at the middle position of the propyl chain. Finally, peak e denotes the methylene protons connected to the R group, which typically represents the polymer backbone or a specific functional group.

Figure 8 shows the ^1^H nuclear magnetic resonance (^1^H NMR) hydrogen spectra of three types of macromolecular silane coupling agents. The peaks at 2.5 ppm and 3.3 ppm correspond to deuterated DMSO and water, respectively. The peaks at 1.96 ppm, 2.78 ppm, and 2.94 ppm are attributed to residual DMAc. The hydrogen peaks e, d, and c of the three methylene groups in the aminopropyl moiety of KH550 are located at 2.62 ppm, 1.43 ppm, and 0.57 ppm, respectively. The peak A at 1.15 ppm and peak B at 3.74 ppm are assigned to the three methyl hydrogen and two methylene hydrogen on the three ethoxy groups, respectively. At the same time, the characteristic peak of 2-aminopyridine disappeared, indicating that KH550 has been grafted onto both ends of the oligomer chains. This result confirms the successful preparation of the macromolecular silane coupling agents [27].

#### 3.1.3. XPS

**Figure 9:**
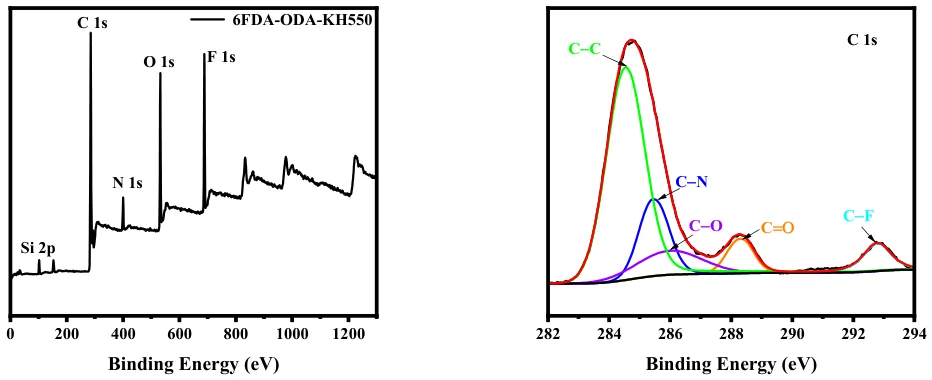

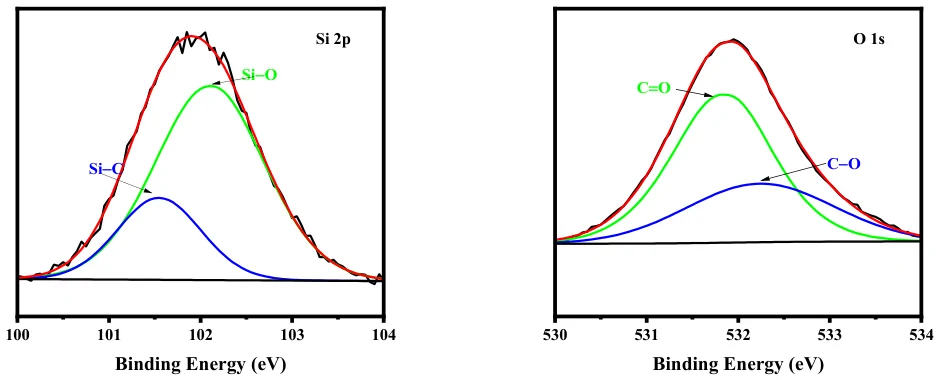
XPS spectra of 6FDA-ODA-KH550 and high-resolution XPS spectra of C1s, O 1s, Si 2p.

Figure 9 shows the XPS spectrum of 6FDA-ODA-KH550, and its results exhibit characteristic peaks at 101.8 eV (Si 2p), 284.8 eV (C 1s), 399.8 eV (N 1s), 531.8 eV (O 1s), and 688.2 eV (F 1s). As shown in Figure 9, the existence form of each element was fitted with subpeak via high resolution XPS spectra. It can be seen from the analysis results that carbon mainly exists in the forms of C−C (284.5 eV), C−N (285.4 eV), C−O (285.9 eV), C=O (288.2 eV), and C−F (292.7 eV) [28]. Oxygen exists mainly in the form C=O (531.8 eV) and C−O (532.2 eV). Silicon exists mainly in the forms Si−C (101.5 eV) and Si=O (102.1 eV) [29]. Nitrogen exists mainly in the form of C−N (399 eV), and fluorine exists mainly in the form of C−F (688 eV).

**Figure 10:**
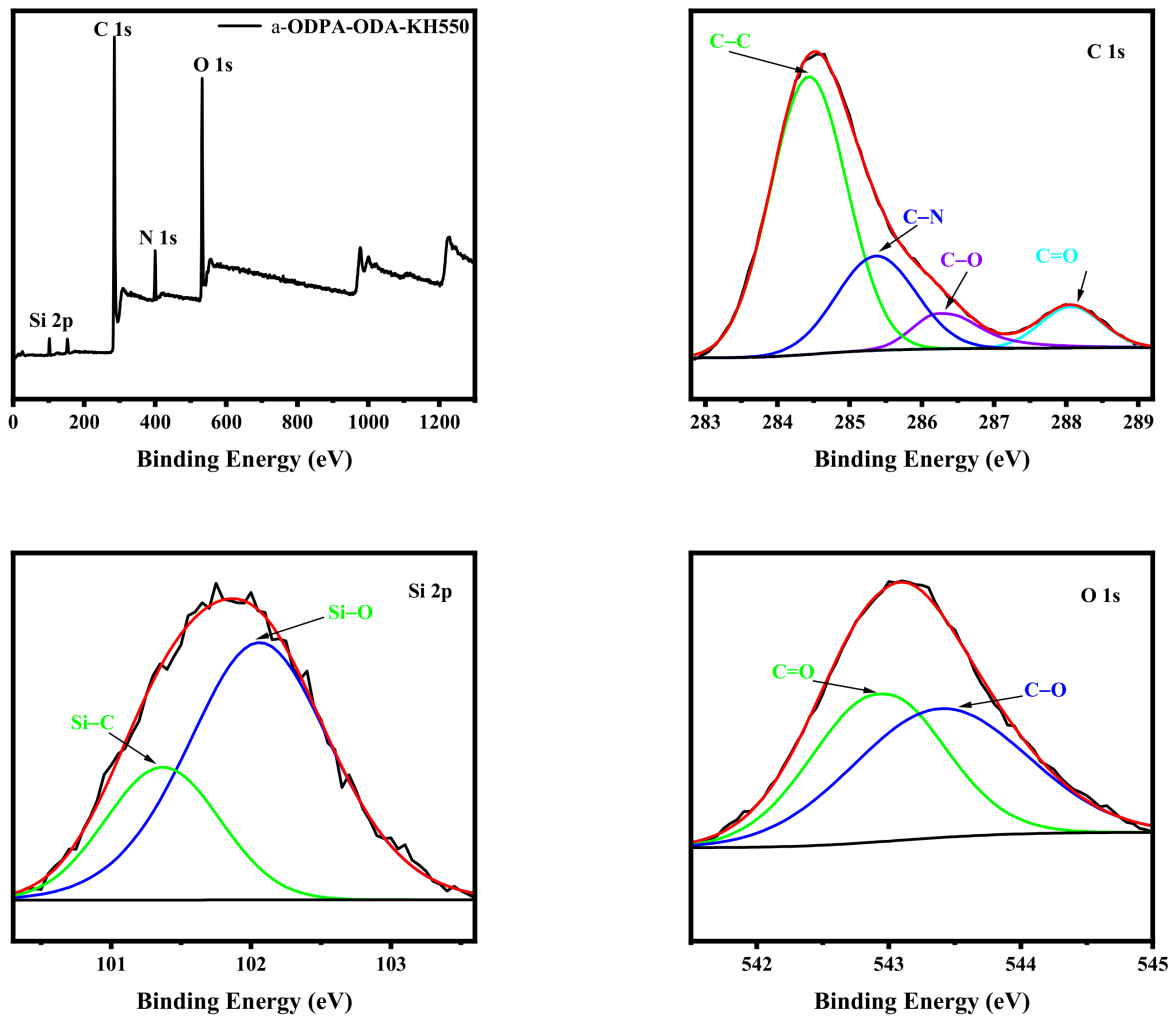
XPS spectra of a-ODPA-ODA-KH550 and high-resolution XPS spectra of C1s, O 1s, Si 2p.

Figure 10 shows the XPS spectrum of a-ODPA-ODA-KH550, and its results exhibit characteristic peaks at 101.9 eV (Si 2p), 284.8 eV (C 1s), 399.8 eV (N 1s), and 531.9 eV (O 1s). As shown in Figure 10, the existence forms of each element were fitted with sub-peak via high resolution XPS spectra. It can be seen from the analysis results that carbon mainly exists in the forms of C−C (284.4 eV), C−N (285.3 eV), C−O (286.2 eV), and C=O (288.0 eV). Oxygen exists mainly in the forms of C=O (531.6 eV) and C−O (533.1 eV). Silicon exists mainly in the forms of Si−C (101.3 eV) and Si=O (102.0 eV). Nitrogen exists mainly in the form of C−N (399.8 eV).

**Figure 11:**
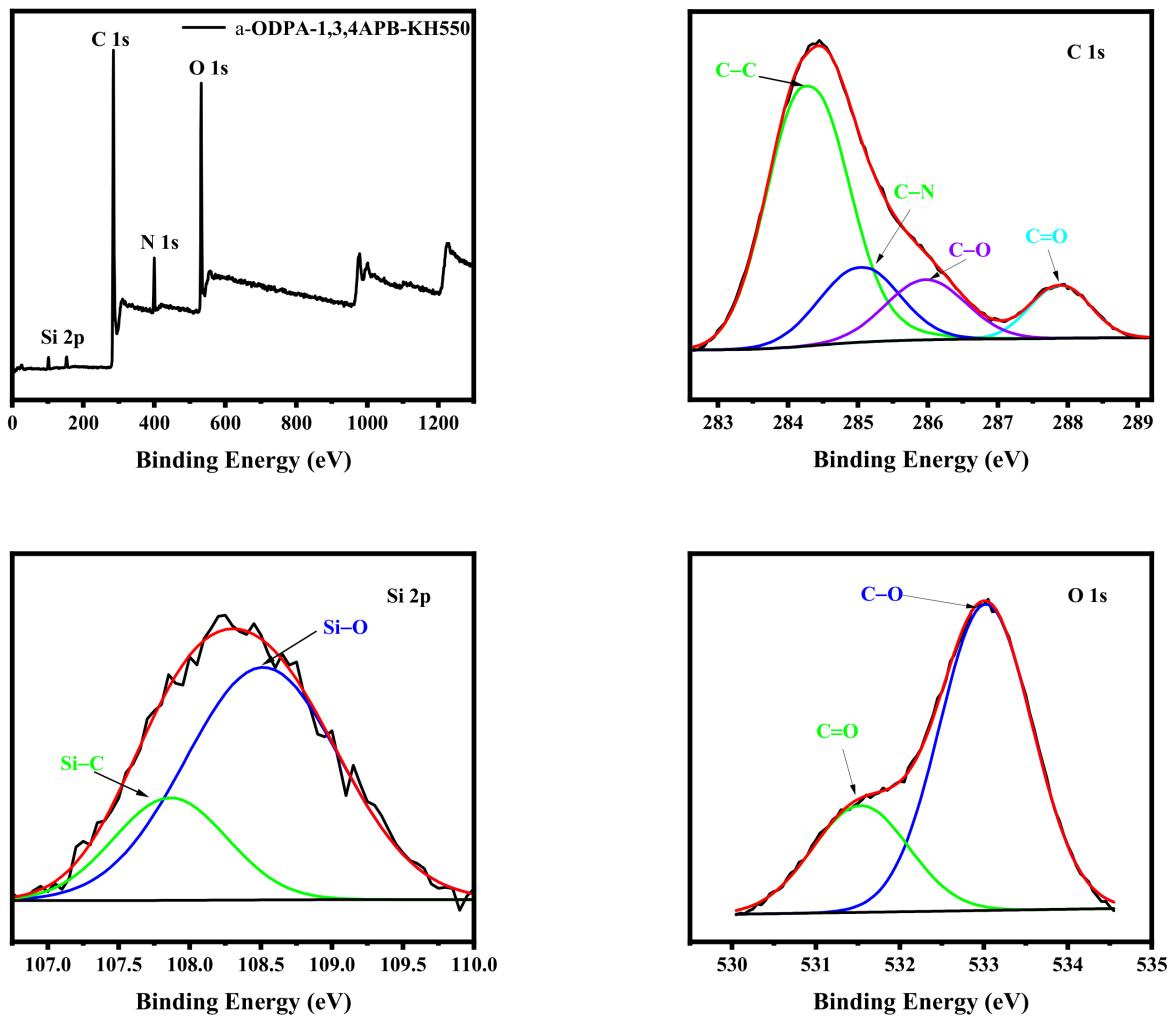
XPS spectra of a-ODPA-1,3,4APB-KH550 and high-resolution XPS spectra of C1s, O 1s, Si 2p.

Figure 11 shows the XPS spectrum of a-ODPA-1,3,4APB -KH550, and its results exhibit characteristic peaks at 101.6 eV (Si 2p), 284.8 eV (C 1s), 399.7 eV (N 1s), and 531.9 eV (O 1s). As shown in Figure 11, the existence forms of each element were fitted with sub-peaks via high resolution XPS spectra. It can be seen from the analysis results that carbon mainly exists in the forms of C−C (284.2 eV), C−N (285.0 eV), C−O (285.9 eV), and C=O (287.8 eV). Oxygen exists mainly in the forms of C=O (531.5 eV) and C−O (533.0 eV). Silicon exists mainly in the forms of Si−C (101.0 eV) and Si=O (101.6 eV). Nitrogen exists mainly in the form of C−N (399.7 eV).

The relative percentage contents of elements of the above three macromolecular silane coupling agents are shown in Table 2. The XPS results show that the Si contents of the three macromolecular silane coupling agents account for 3.29%, 3.37%, and 3.66%, respectively, indicating that the macromolecular silane coupling agents have been successfully prepared [30].

### 3.2. Thermal Stability Analysis

**Figure 12:**
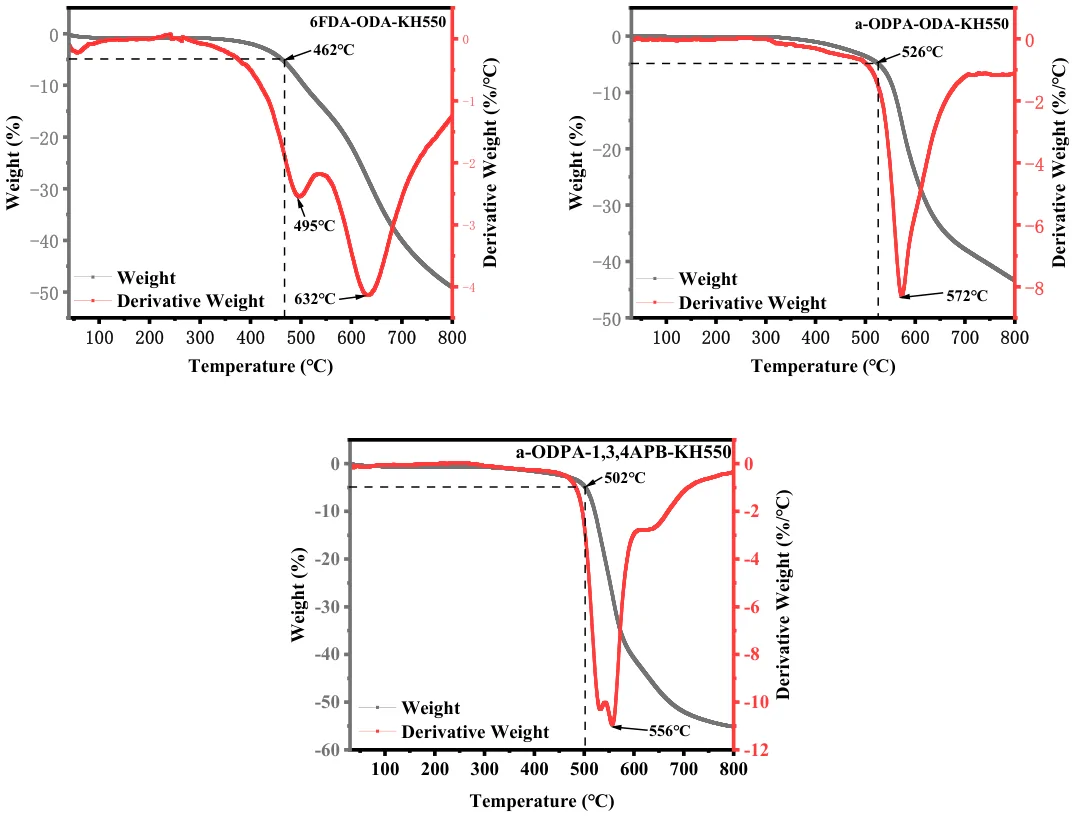
TG and DTG diagrams of 6FDA−ODA−KH550, a-ODPA−ODA−KH550, and a−ODPA−1,3,4APB−KH550.

Figure 12 presents the TG and DTG curves of the three synthesized macromolecular silane coupling agents (MSCAs). The 5% thermal weight loss temperatures (T5%) of 6FDA-ODA-KH550, a-ODPA-ODA-KH550, and a-ODPA-1,3,4APB-KH550 are 462 °C, 526 °C, and 502 °C, respectively, all of which are far higher than the typical processing temperature (~360 °C) of polyimide composites. This confirms that the MSCAs will not decompose during extrusion granulation and molding, ensuring the stable performance of the final composites.

To clarify the thermal decomposition mechanism, peak fitting of the DTG curves was performed, and the results were compared with the reported thermal degradation behavior of polyimide-based materials [31,32]. As shown in the fitted DTG curves, 6FDA-ODA-KH550 exhibits two distinct degradation stages: the first stage centered at ~495 °C, corresponding to the pyrolysis of the imide ring and the side chain silane coupling groups (–Si(OEt)_3_), with a mass loss of ~35%, consistent with the reported degradation of polyimide oligomers; the second stage centered at ~632 °C, attributed to the degradation of the aromatic main chain and the –CF_3_-containing linking group, with a mass loss of ~60%. The high thermal stability of 6FDA-ODA-KH550 originates from its all aromatic backbone and the electron-withdrawing –C(CF_3_)_2_– group, which enhances the bond energy of the main chain, as verified by similar polyimide systems.

For a-ODPA-ODA-KH550, the DTG curve shows a single dominant degradation peak at ~572 °C, with a narrow decomposition interval, indicating a synchronous degradation of the ether bond-containing main chain and imide rings. In contrast, a-ODPA-1,3,4APB-KH550 presents a broad two-stage DTG peak (468–607 °C), which is fitted into two sub-peaks centered at ~485 °C and ~580 °C. The first subpeak corresponds to the cleavage of amide bonds and ether bonds in the main chain, while the second subpeak is attributed to the pyrolysis of the aromatic backbone, generating volatile products such as CO_2_, CH_4_, and HCN, which is consistent with the thermal decomposition mechanism of ether-containing polyimides.

In summary, the TG/DTG results, combined with DTG peak fitting and literature comparison, systematically reveal the thermal decomposition mechanism of the three MSCAs, confirming their excellent thermal stability suitable for high temperature processing of polyimide composites.

### 3.3. Gel Permeation Chromatography

In order to determine the molecular weight of three macromolecular coupling agents, gel permeation chromatography (GPC) tests were performed. The data shown in Table 3 were obtained using different statistical methods to calculate molecular weights, and the actual molecular weights were approximately normally distributed. The table shows that the average molecular weights of 6FDA-ODA-KH550, a-ODPA-ODA-KH550, and A-ODPA-1,3,4APB-KH550 are 3504 g/mol, 2629 g/mol, and 2635 g/mol, respectively. It was found that the average molecular weights measured in the experiment were slightly larger than the theoretically calculated values. This may be due to the fact that the intermediate used in the experiment had higher reactivity with diamine at the reaction temperature, and a longer molecular chain was generated in the reaction process than that calculated in theory.

### 3.4. Solubility Analysis

To achieve a better surface treatment effect of the three macromolecular silane coupling agents on fibers, their solubility at room temperature was tested. In general, polyimide molecules containing N-pentaclactam exhibit a highly regular and symmetrical five-membered ring structure with high rigidity. Ordinary solvents are unable to penetrate their molecular interior, making them insoluble in common solvents. Table 4 shows the solubility results of three macromolecular coupling agents. They can be dissolved in most polar organic solvents, such as N, N-dimethylacetamide, N, N-dimethylformamide, and N-methyl pyrrolidone. They also show good solubility in many common organic solvents including tetrahydrofuran and chloroform, but exhibit low solubility in toluene and are completely insoluble in ethanol. This can be explained as follows: the strong polarity of the trifluoromethyl group in 6FDA increases the overall polarity of the polymer, which favors its solubility in polar solvents. In addition, the bulky trifluoromethyl groups enlarge the free volume of the polymer, reduce chain packing density, and facilitate the penetration of solvent molecules, thereby enhancing the solubility at room temperature. Meanwhile, a-ODPA introduces an asymmetric five membered ring structure, which lowers the symmetry of the polyimide backbone and further improves solubility. The above results show that the three macromolecular silane coupling agents can be dissolved in polar solvents and thus suitable for fiber surface treatment.

The above solubility characteristics are beneficial to the composite preparation process and final performance. Good solubility in polar solvents ensures that the macromolecular silane coupling agents can be fully dissolved to form a uniform treatment solution, which is conducive to the full wetting and coating on the fiber surface during solution treatment. The uniform interfacial layer formed on fibers helps to improve interfacial compatibility and bonding strength between fibers and the matrix, promotes effective stress transfer, and thus optimizes the mechanical properties and service stability of the composites.

### 3.5. Mechanical Property Characterization

Table 5 shows the test results for composites prepared using different coupling agents and different fibers. The tensile strengths of YGM-GF, YGM-3GF, YGM-CF, and YGM-3CF are 77.3 MPa, 88.3 MPa, 90.9 MPa, and 101.7 MPa, respectively. It can be seen that the strength of carbon fiber/polyimide composites is higher than that of glass fiber/polyimide composites, and the strength of 3 mm fiber/polyimide composites is higher than that of fiber powder/polyimide composites. This is because carbon fiber has stronger rigidity than glass fiber, and 3 mm fiber has a higher length-to-diameter ratio than fiber powder, so they play a better reinforcement effect. The surface of the same fiber also has different strengthening effects after grafting with different coupling agents. For example, in the glass fiber powder/YGM composite system, the tensile strength of the glass fiber powder reinforced polyimide without coupling agent treatment is only 77.3 MPa, while that treated with the small molecule silane coupling agent KH550 increases to 79.1 MPa. After treatment with macromolecular silane coupling agent, the tensile strength can reach a maximum of 87.1 MPa, showing a significant enhancement effect. In all the test samples, the average tensile strength and bending strength of the composite after the treatment of small molecule silane coupling agent increased by 3.9% and 4.0%, whereas those treated with macromolecular silane coupling agents increased by 11.0% and 9.8%, respectively.

This is due to the poor heat resistance of KH550, which evaporates out of the system and even decomposes during the processing of the material. Although there is a small amount of enhancement, the enhancement effect is limited. Compared with the three macromolecular silane coupling agents, the commercial KH550 exhibits inferior high temperature stability. KH550 has a boiling point of 217 °C and an initial thermal decomposition temperature of ~220 °C, which are far below the processing temperature of PEEK composites (300–380 °C). During high temperature molding, unreacted KH550 physically adsorbed on fiber surfaces tends to volatilize significantly, resulting in an incomplete interfacial layer and thus limited improvement in composite mechanical properties. In contrast, the macromolecular silane coupling agents with rigid polyimide backbones possess much higher thermal stability, enabling stable interfacial modification during composite processing [33].

In contrast, the three macromolecular silane coupling agents synthesized in this work possess excellent thermal stability. Most of them remain intact without loss or decomposition during extrusion and molding after fiber grafting. Furthermore, the backbone of these coupling agents is polyimide, which has a molecular structure similar to that of the matrix resin. This not only facilitates uniform dispersion in the matrix but also provides stronger interfacial bonding, enabling more effective stress transfer across the fiber–matrix interface. As a result, the fibers fully exert their load-bearing role in the composite, leading to further improved rigidity and significantly enhanced mechanical properties. Compared with the small molecule silane coupling agents, the macromolecule silane coupling agents have a longer molecular chain, which can realize the chain entanglement between the coupling agent chain segment on the fiber surface and the matrix resin [19]. Consequently, composites reinforced with fibers treated with macromolecular coupling agents exhibited superior mechanical properties compared to those treated with small molecule coupling agents.

**Figure 13:**
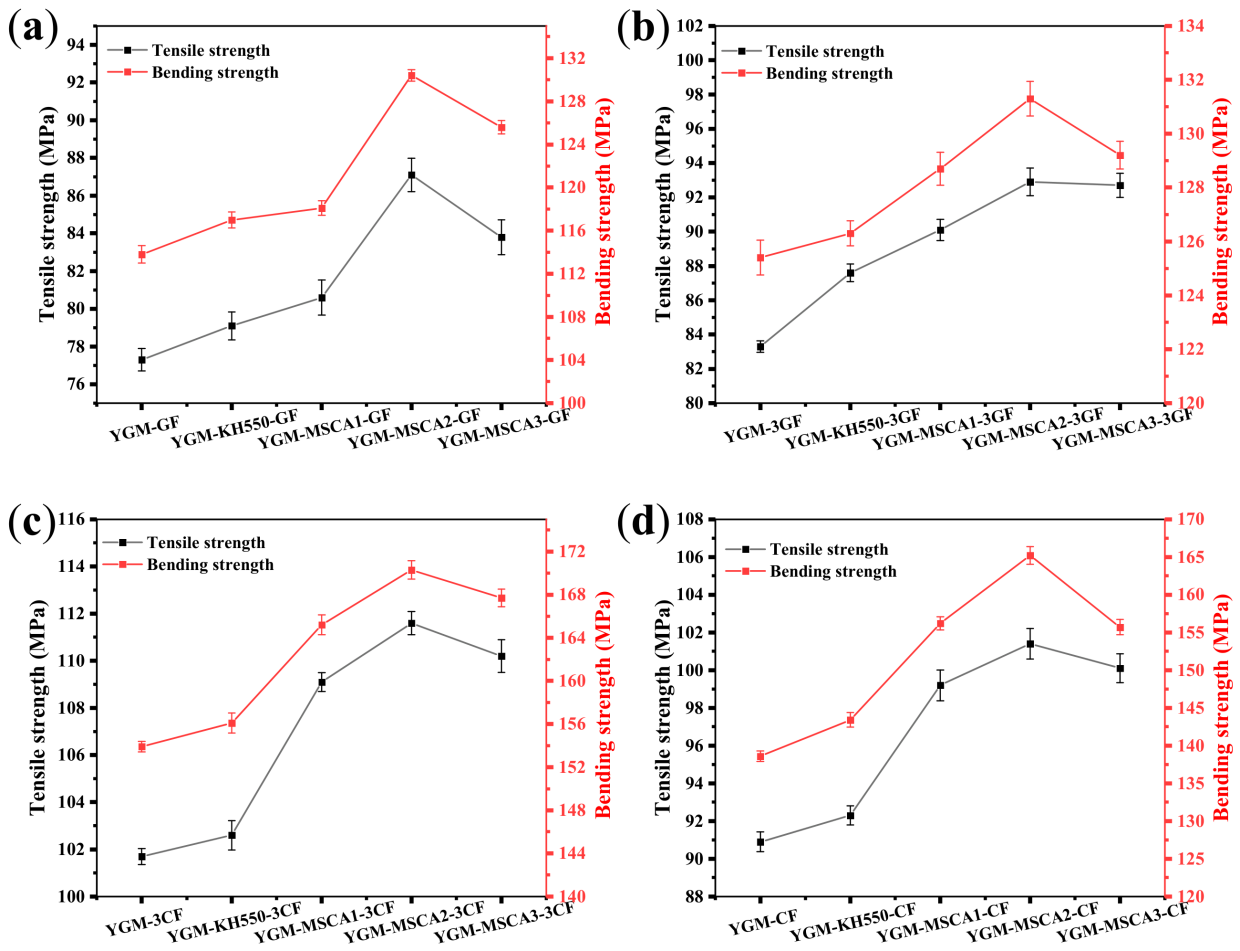
(**a**) Tensile bending strength diagram of GM-GF treated with different coupling agents. (**b**) Tensile bending strength diagram of GM-3GF treated with different coupling agents. (**c**) Tensile bending strength diagram of YGM-CF treated with different coupling agents. (**d**) Tensile bending strength diagram of YGM-3CF treated with different coupling agents.

**Figure 14:**
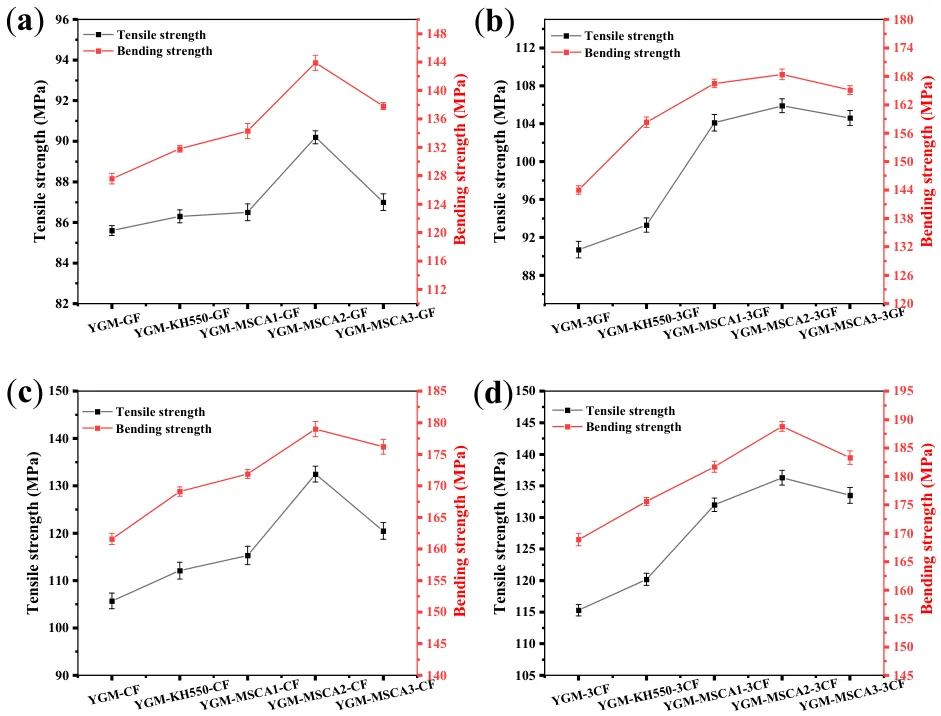
(**a**) Tensile bending strength diagram of PI2542-GF treated with different coupling agents. (**b**) Tensile bending strength diagram of PI2542-3GF treated with different coupling agents. (**c**) Tensile bending strength diagram of PI2542-CF treated with different coupling agents. (**d**) Tensile bending strength diagram of PI2542-3CF treated with different coupling agents.

Figure 13 and Figure 14 show the mechanical properties of fiber-reinforced YGM and PI2542 polyimide composites, respectively. By comparing composites reinforced with fibers treated with three macromolecular silane coupling agents, it was found that the highest tensile and bending strengths of the MSCA1-treated composites were 132.0 MPa and 181.7 MPa, respectively. For PI2542-MSCA2-3CF treated with MSCA2, the tensile strength and bending strength reached 136.3 MPa and 188.8 MPa, respectively, representing the best reinforcing effect among the three macromolecular silane coupling agents. The composite with optimal performance treated with MSCA3 was PI2542-MSCA3-3CF, with tensile and bending strengths of 133.5 MPa and 183.3 MPa. This is because the structure of MSCA2 and MSCA3 is ether anhydride/ether amine, and the matrix material is also ether anhydride/ether amine structure. Such structural similarity gives rise to similar van der Waals interactions between the coupling agents and the matrix, allowing the macromolecular coupling agents on the fiber surface to entangle more easily with the matrix chains.

The results of differential scanning calorimetry (DSC) and static mechanical tests perfectly corroborated the core interfacial reinforcement mechanism typically identified in dynamic mechanical analysis (DMA) characterizations [34]. DSC data indicated that the average glass transition temperature (T_g_) of the composites treated with the macromolecular silane coupling agent (MSCA) increased by 2.3 °C. This microscopically confirmed that the long molecular chains of MSCA diffused and entangled with the polyimide matrix, effectively restricting the mobility of polymer segments. This restriction represented the underlying physical mechanism for the broadening of the damping peak (tan δ) and its shift toward higher temperatures observed in DMA [35].

Meanwhile, the macroscopic mechanical improvements validated the logic behind the enhancement of storage modulus (E’) in DMA tests [36]. Results showed that MSCA modification increased the average tensile and flexural strengths of the composites by 11.0% and 9.8%, respectively, with the maximum tensile strength reaching 136.3 MPa. This significant enhancement was attributed to the MSCA’s rigid polyimide backbone, which was structurally similar to the matrix and acted as a “molecular bridge” at the interface [37]. The robust interfacial bonding created by long chain entanglement facilitated efficient stress transfer between the matrix and fibers, serving as the fundamental reason for the substantial retention of deformation resistance (i.e., storage modulus) during high temperature dynamic mechanical testing.

### 3.6. Morphology Characterization

**Figure 15:**
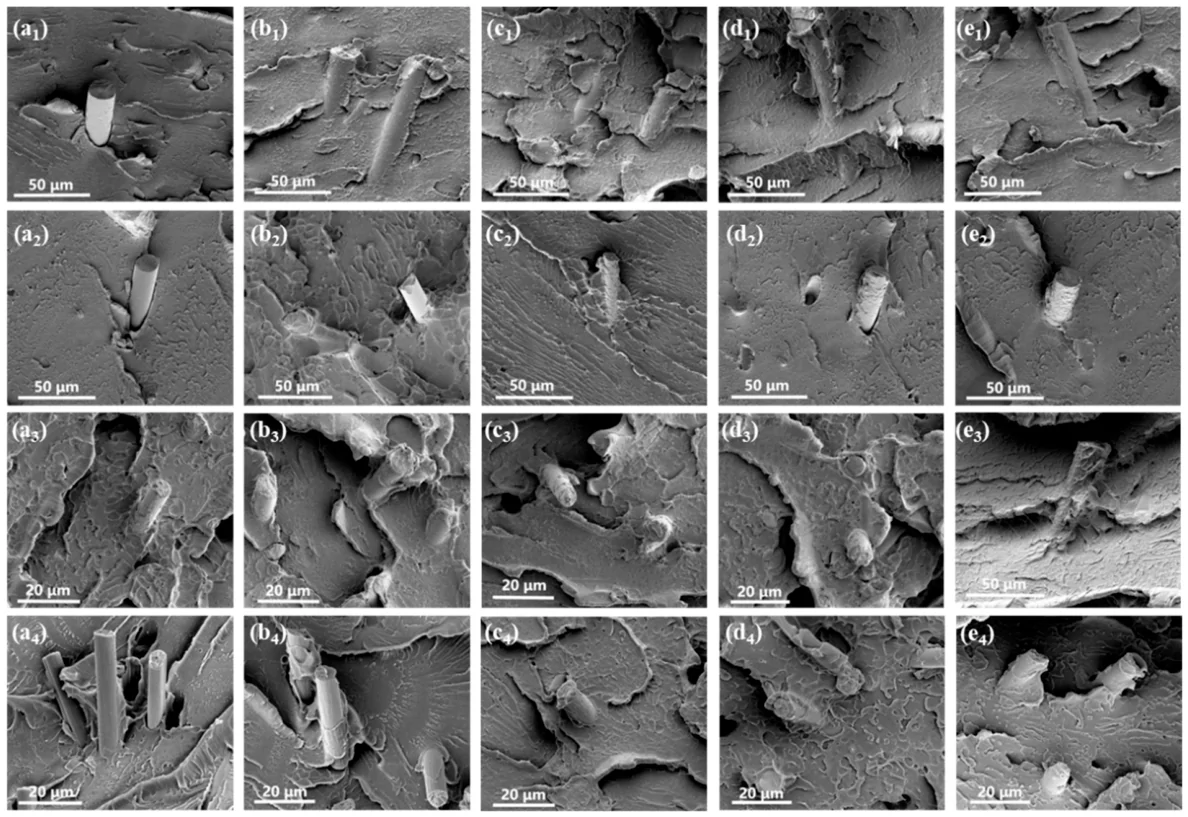
(**a**–**e**) Section SEM images of untreated, KH550-treated, MSCA1-treated, MSCA2-treated, and MSCA3-treated fiber-reinforced composites, respectively. (**1**–**4**) SEM images of the cross sections of PI2542-GF, PI2542-3GF, PI2542-CF, and PI2542-3CF composites under the same coupling agent.

Interfacial compatibility between the two phases can also be directly evaluated by observing the cross-sectional morphology of the composites. Figure 15 shows SEM images of the fracture surfaces of fiber-reinforced PI2542 polyimide resin composites treated with different coupling agents. It can be seen from the images that the fibers are well dispersed in the matrix and no serious agglomeration phenomenon occurs. The fibers in Figure 15a are not treated, and it can be seen that the fibers are directly pulled out from the matrix upon fracture, with relatively long pullout lengths. The surface of the glass fibers remains smooth, the carbon fiber ravings are clearly visible, and almost no matrix resin is left on the fiber surfaces. The brittle fracture is manifested, and there is an obvious gap between the fibers and the matrix, indicating that the two phase interface adhesion between the fibers and the PI matrix is poor. The fibers are simply embedded in the PI matrix. Therefore, when PI/fibril composites are subjected to external forces, the fibers are easily pulled out, making the cross section of the material have obvious defects, such as holes of different sizes. Figure 15b shows that after the treatment with small molecule silane coupling agent KH550, there is little resin adhesion on the surface of the exposed fibers, and the two phase interface is still clearly visible, indicating that the binding between the KH550-modified fibers and the PI matrix is still weak. In Figure 15c–e, after treatment with macromolecular silane coupling agent, it is obvious that the surface of the fibers is covered with a layer of resin, and most of the fibers do not unstick, showing toughness fracture, indicating that the macromolecular silane coupling agent improves the compatibility of the fibers and the resin matrix, and the treated fibers are evenly distributed in the PI matrix, and exhibit the best interfacial bonding with the matrix.

### 3.7. Thermal Performance Characterization

**Figure 16:**
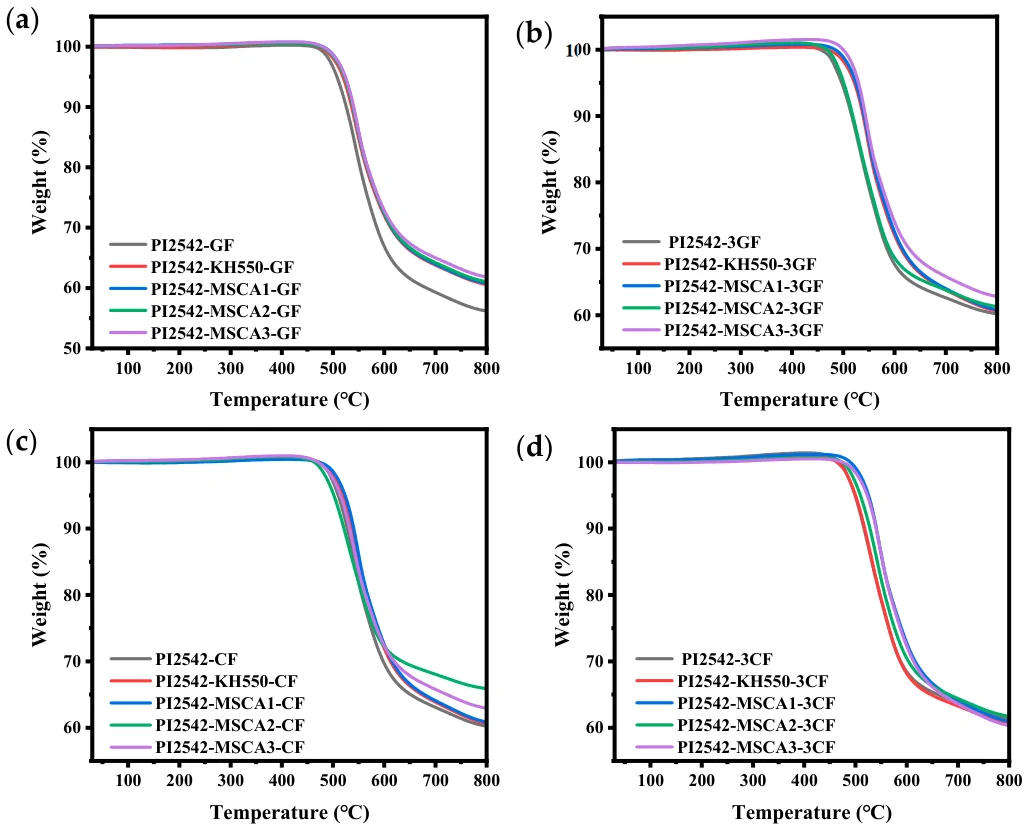
(**a**) Thermogravigram of PI2542-GF treated with different coupling agents. (**b**) Thermogravigram of PI2542-3GF treated with different coupling agents. (**c**) Thermogravigram of PI2542-CF treated with different coupling agents. (**d**) Thermogravigram of PI2542-3GF treated with different coupling agents.

Thermal properties are one of the important parameters of polymer composites, especially in the field of aerospace as heat resistant materials. Figure 16 shows the TGA curves of different fiber-reinforced polyimide composites from 30 °C to 800 °C. The corresponding data of T5, T10 (weight loss temperature of 5% and 10%), and RM% (residual mass percentage at 800 °C) are summarized in Table 6. As can be seen from the table, the highest temperature of PI2542-KH550-fiber at T5 and T10 reaches 521.6 °C and 538.6 °C, respectively, while the highest temperature of PI2542-MSCA-fiber at T5 and T10 reaches 529.6 °C and 543.4 °C, respectively. The fiber composites treated with macromolecular silane coupling agent showed higher stability. This is because the end of the macromolecular coupling agent is connected to the fiber surface, and the macromolecular silane coupling agent chains entangle with the PI long chains, hindering the thermal movement of the PI molecular chains. In addition, energy can be rapidly transferred, dissipated, or absorbed through the fibers, thereby improving the performance of the PI composite material. In conclusion, the introduction of macromolecular silane coupling agents enables the construction of a high quality organic–inorganic interface, providing an effective strategy for enhancing the thermal properties of composite materials.

**Figure 17:**
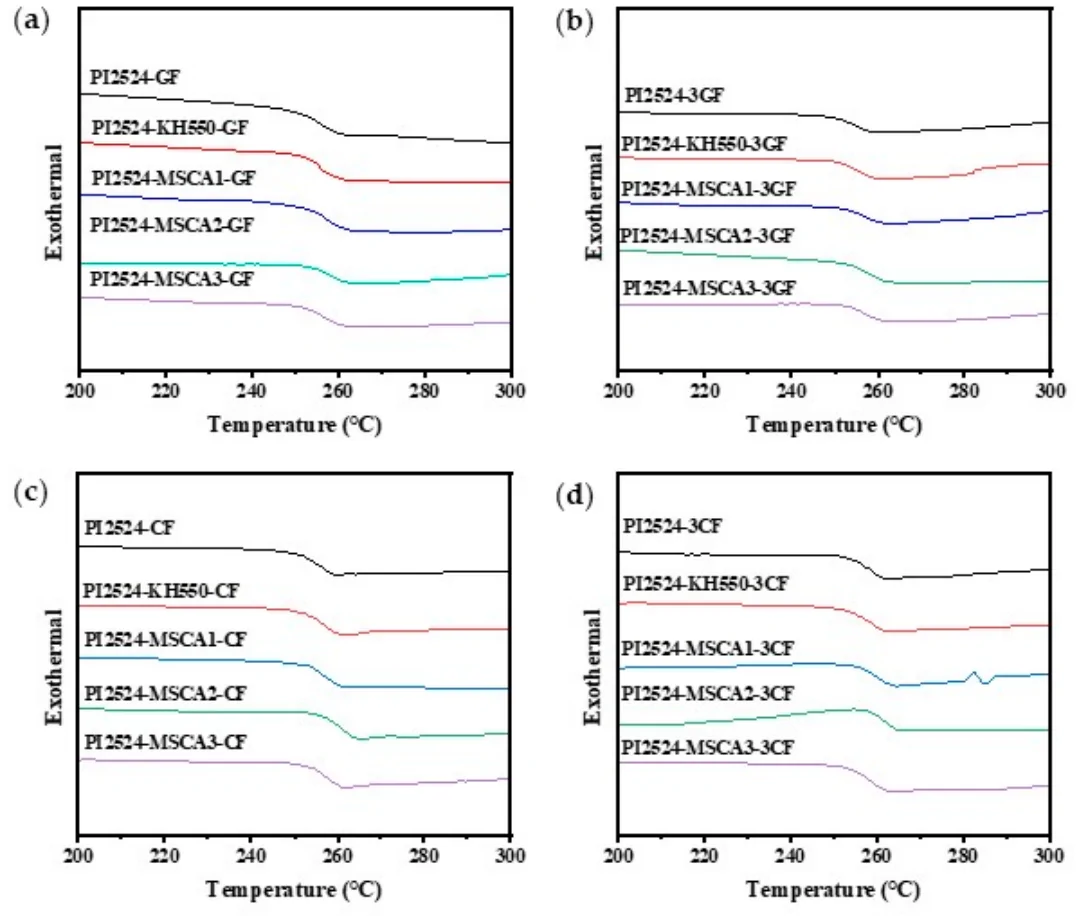
(**a**) DSC images of PI2542-GF treated with different coupling agents. (**b**) DSC images of PI2542-3GF treated with different coupling agents. (**c**) DSC images of PI2542-CF treated with different coupling agents. (**d**) DSC images of PI2542-3GF treated with different coupling agents.

The rigidity of the molecular chain is mainly measured by the glass transition temperature T_g_. In general, the stronger the rigidity of the molecular chain, the higher the glass transition temperature, and the smaller the free volume of the polymer that can be utilized by thermal motion. In polyimide resins, the segments become entangled due to the van der Waals forces between themselves, and this entanglement is one of the key factors hindering their fluidity. As the fibers are added to the polyimide resin, the mobility of the molecular segments is hindered or restricted by the rigid fibers; thus, the T_g_ of the resin increases after fiber reinforcement. Figure 17 shows the DSC images of four kinds of fibers treated with different coupling agents. Table 6 shows their respective glass transition temperatures. The results show that the glass transition temperature of composites without any coupling agent treatment is the lowest. For example, the T_g_ of PI2542-3CF is 256.2 °C, and the T_g_ of PI2542-3CF is slightly increased to 256.9 °C after small molecule coupling agent treatment. In contrast, the average T_g_ of composites treated with macromolecular silane coupling agents increases to 258.5 °C, 260.0 °C, and 258.4 °C, respectively. In comprehensive comparison, the T_g_ of the fiber/PI2542 composite treated with KH550 increased by 0.7 °C on average, while that of the fiber/PI2542 composite treated with macromolecular silane coupling agent increased by 2.3 °C on average.

It should be noted that slight fluctuations in T5 values among different samples are within the normal range of experimental error, which may result from minor differences in sample preparation, fiber dispersion, and testing conditions. These small inconsistencies do not affect the general trend of improved thermal stability.

Due to the short molecular chain of KH550, the modified fiber cannot form effective bonding with the polyimide resin. The binding force between the fibers and the polyimide resin phase is also mainly van der Waals force, which is similar to the binding force between the polyimide resin chain segments themselves. Therefore, the T_g_ of fiber treated with KH550 can be enhanced, but the effect is limited.

Macromolecular silane coupling agents have long molecular chains, which can diffuse and entangle with polyimide polymer chains, forming an interface layer between fiber and polyimide matrix. There is a strong interaction between the fiber treated with macromolecular silane coupling agent and polyimide resin, and this interaction hinders the migration of polyimide resin segments. This results in lower segment mobility at the same temperature. Therefore, the T_g_ of fiber/polyimide composites treated with macromolecular silane coupling agent increased significantly.

The fiber-reinforced polyimide (PI) composites modified by macromolecular silane coupling agents (MSCAs) prepared in this work exhibit excellent comprehensive performance, with a tensile strength of 136.3 MPa and a T10 (temperature at 10% mass loss) of 543.4 °C. Compared with existing PI materials, this system shows obvious advantages in both mechanical and thermal properties. Specifically, its tensile strength is 13.6–45.2% higher than that of conventional pure PI (94–120 MPa) and 13.6–29.8% higher than that of PI composites modified by traditional small molecule silane coupling agents (e.g., KH550, 105–120 MPa), which is attributed to the stable interfacial bonding formed by the “molecular bridge” effect of MSCA—consistent with the interfacial enhancement mechanism reported [38]. In terms of thermal stability, the T10 of 543.4 °C is at the medium to high level among pure PI systems (500–540 °C) and 3–8 °C higher than that of small-molecule silane modified systems, benefiting from the excellent thermal stability of the polyimide rigid segment and Si–O bonds in MSCA, as verified. Compared with nanomodified PI systems, this MSCA-modified composite avoids the problems of filler agglomeration and poor interfacial stability, while maintaining a simple preparation process and high performance stability—an advantage that outperforms the nano-filler-modified PI composites reported [39]. These findings are supported by relevant recent studies, and the synergistic improvement of mechanical and thermal properties of this composite breaks the bottleneck of traditional modification, showing broad application prospects in aerospace and high-end equipment fields requiring long term high temperature service.

## 4. Conclusions

In this work, 6FDA-ODA-KH550, a-ODPA-ODA-KH550, and a-ODPA-1,3,4APB-KH550 macromolecular silane coupling agents were synthesized via chemical imidization and transamidation reactions. The chemical structures were confirmed by FTIR, NMR, and XPS analyses, which collectively demonstrated the successful incorporation of KH550 into the polyimide oligomer backbone. The synthesized macromolecular coupling agents exhibited excellent thermal stability, with decomposition temperatures well above the processing temperature of polyimide composites, ensuring their structural integrity during composite fabrication. They also showed good solubility in common polar organic solvents, facilitating their application in fiber surface treatment. When applied to modify the surface of glass and carbon fibers, these macromolecular coupling agents demonstrated a superior reinforcing effect compared to the conventional small molecule KH550. The enhanced mechanical properties were attributed to the formation of strong interfacial interactions, including chain entanglement between the long polyimide-based chains of the coupling agent and the polyimide matrix. The SEM observations confirmed that the macromolecular coupling agents promoted resin adhesion to the fiber surface, resulting in a tough fracture mode, in contrast to the smooth fiber surfaces and brittle fracture observed for untreated or KH550-treated samples. Furthermore, composites modified with the macromolecular coupling agents exhibited improved thermal stability and increased glass transition temperatures, reflecting enhanced interfacial adhesion that restricts polymer chain mobility. In summary, this work provides an effective strategy for synthesizing thermally stable macromolecular silane coupling agents. By improving interfacial compatibility between fibers and the polyimide matrix, these agents significantly enhance the mechanical and thermal properties of fiber-reinforced polyimide composites, offering a promising approach for developing high performance composite materials.

## Figures and Tables

**Table 1:** The experimental procedures, raw materials, and yields for the preparation of the three macromolecular silane coupling agents.

Target Product	Optimization Variables	Reagent Types and Dosages	Reaction Conditions	Yield (%)
6FDA-ODA-KH550	Polyimide precursor (6FDA-ODA-2APY) synthesis.	ODA: 6.00 g (0.03 mol), 6FDA: 17.76 g (0.04 mol), DMAc: 190 g, 2-aminopyridine: 1.88 g, Acetic anhydride: 37.02 g (0.36 mol), Triethylamine: 36.42 g (0.36 mol).	Room temperature, N_2_ atmosphere, 4 h (polyamide acid), 2 h (end-capping), 24 h (imidization), Precipitation with 5× ethanol, 80 °C vacuum drying for 24 h.	88.6
	Transamidation reaction (with KH550).	6FDA-ODA-2APY: 24.2 g (0.01 mol), DMAc: 300 mL, KH550: 6.63 g (0.03 mol), Anhydrous ethanol (precipitation).	Room temperature dissolution (5 min), 140 °C heating reflux for 24 h, Centrifugation (3 times), 80 °C vacuum drying for 24 h.	85.3
	Optimal condition (integrated).	As above (precursor + transamidation reagents).	Integrated reaction conditions of precursor synthesis and transamidation.	84.1 (overall yield)
a-ODPA-ODA-KH550	Polyimide precursor (a-ODPA-ODA-2APY) synthesis.	ODA: 6.00 g (0.03 mol), a-ODPA: 12.4 g (0.04 mol), DMAc: 190 g, 2-aminopyridine: 1.88 g, Acetic anhydride: 37.02 g (0.36 mol), Triethylamine: 36.42 g (0.36 mol).	Room temperature, N_2_ atmosphere, 4 h (polyamide acid), 2 h (end-capping), 24 h (imidization), Precipitation with 5× ethanol, 80 °C vacuum drying for 24 h.	90.2
	Transamidation reaction (with KH550).	a-ODPA-ODA-2APY: 18.8 g (0.01 mol), DMAc: 300 mL, KH550: 6.63 g (0.03 mol), Anhydrous ethanol (precipitation).	Room temperature dissolution (5 min), 140 °C heating reflux for 24 h, Centrifugation (3 times), 80 °C vacuum drying for 24 h.	86.7
	Optimal condition (integrated).	As above (precursor + transamidation reagents).	Integrated reaction conditions of precursor synthesis and transamidation.	85.9 (overall yield)
a-ODPA-1,3,4APB-KH550	Polyimide precursor (a-ODPA-1,3,4APB-2APY) synthesis.	1,3,4APB: 8.76 g (0.03 mol), a-ODPA: 12.4 g (0.04 mol), DMAc: 190 g, 2-aminopyridine: 1.88 g, Acetic anhydride: 37.02 g (0.36 mol), Triethylamine: 36.42 g (0.36 mol).	Room temperature, N_2_ atmosphere, 4 h (polyamide acid), 2 h (end-capping), 24 h (imidization), Precipitation with 5× ethanol, 80 °C vacuum drying for 24 h.	89.5
	Transamidation reaction (with KH550).	a-ODPA-1,3,4APB-2APY: 20.4 g (0.01 mol), DMAc: 300 mL, KH550: 6.63 g (0.03 mol), Anhydrous ethanol (precipitation).	Room temperature dissolution (5 min), 140 °C heating reflux for 24 h, Centrifugation (3 times), 80 °C vacuum drying for 24 h.	87.1
	Optimal condition (integrated).	As above (precursor + transamidation reagents).	Integrated reaction conditions of precursor synthesis and transamidation.	86.3 (overall yield)

**Table 2:** The types and relative contents of elements contained in macromolecular silane coupling agents.

Sample	Element	Energy	Relative Content%
6FDA-ODA-KH550	Si	101.8 eV	3.29%
	C	284.8 eV	65.18%
	N	399.8 eV	5.13%
	O	531.8 eV	14.69%
	F	688.2 eV	11.71%
a-ODPA-ODA-KH550	Si	106.5 eV	3.37%
	C	284.8 eV	71.55%
	N	399.8 eV	6.54%
	O	531.9 eV	18.54%
a-ODPA-1,3,4APB-KH550	Si	101.6 eV	2.66%
	C	284.8 eV	71.71%
	N	399.7 eV	6.85%
	O	531.9 eV	18.78%

**Table 3:** GPC of three macromolecular silane coupling agents.

Sample	Theoretical Molecular Weight	Peak Molecular Weight	Numerical Mean Molecular Weight	Weight Average Molecular Weight	z Average Molecular Weight	z + 1 Average Molecular Weight	Polydispersity
6FDA-ODA-KH550	2674	7140	3504	7932	13,711	20,113	2.26
a-ODPA-ODA-KH550	2138	7926	2629	8868	19,577	37,831	3.37
a-ODPA-1,3,4APB-KH550	2294	6254	2635	6832	12,173	17,591	2.59

**Table 4:** The ease of dissolution of 6FDA-ODA-KH550, a-ODPA-ODA-KH550, and a-ODPA-1,3,4APB-KH550 in different solvents.

Sample	DMAc	DMF	DMSO	NMP	Toluene	THF	CHCl_3_	Acetone	EtOH
6FDA-ODA-KH550	++	++	++	++	+	++	++	++	-
a-ODPA-ODA-KH550	++	++	++	++	+	++	++	++	-
a-ODPA-1,3,4APB-KH550	++	++	++	++	+	++	++	++	-

++: easy to dissolve. +: partial dissolution. -: does not dissolve.

**Table 5:** Mechanical properties of polyimide composites treated with fibers with different coupling agents.

Sample	Tensile Strength (MPa)	Bending Strength (MPa)	Modulus of Elasticity (GPa)	Bending Modulus (GPa)
YGM-GF	77.3 ± 0.595	113.8 ± 0.808	2.0 ± 0.022	3.1 ± 0.054
YGM-KH550-GF	79.1 ± 0.741	117.0 ± 0.744	2.1 ± 0.083	3.2 ± 0.057
YGM-MSCA1-GF	80.6 ± 0.934	118.1 ± 0.676	2.1 ± 0.090	3.2 ± 0.065
YGM-MSCA2-GF	87.1 ± 0.883	130.4 ± 0.538	2.2 ± 0.081	3.4 ± 0.039
YGM-MSCA3-GF	83.8 ± 0.923	125.6 ± 0.624	2.2 ± 0.023	3.3 ± 0.045
YGM-3GF	83.3 ± 0.338	125.4 ± 0.650	2.2 ± 0.059	3.3 ± 0.036
YGM-KH550-3GF	87.6 ± 0.514	126.3 ± 0.466	2.2 ± 0.091	3.3 ± 0.069
YGM-MSCA1-3GF	90.1 ± 0.619	128.7 ± 0.612	2.2 ± 0.055	3.4 ± 0.026
YGM-MSCA2-3GF	92.9 ± 0.805	131.3 ± 0.642	2.3 ± 0.078	3.4 ± 0.093
YGM-MSCA3-3GF	92.7 ± 0.699	129.2 ± 0.516	2.3 ± 0.034	3.4 ± 0.017
YGM-CF	90.9 ± 0.522	138.6 ± 0.692	2.5 ± 0.088	3.8 ± 0.021
YGM-KH550-CF	92.3 ± 0.506	143.4 ± 0.970	2.5 ± 0.062	3.9 ± 0.080
YGM-MSCA1-CF	99.2 ± 0.818	156.2 ± 0.872	2.7 ± 0.045	4.2 ± 0.095
YGM-MSCA2-CF	101.4 ± 0.810	165.2 ± 1.158	2.9 ± 0.067	4.5 ± 0.012
YGM-MSCA3-CF	100.1 ± 0.773	155.7 ± 1.014	2.8 ± 0.036	4.3 ± 0.020
YGM-3CF	101.7 ± 0.339	153.9 ± 0.484	2.7 ± 0.061	4.1 ± 0.065
YGM-KH550-3CF	102.6 ± 0.620	156.1 ± 0.920	2.7 ± 0.042	4.1 ± 0.091
YGM-MSCA1-3CF	109.1 ± 0.401	165.2 ± 0.923	3.0 ± 0.074	4.4 ± 0.048
YGM-MSCA2-3CF	111.6 ± 0.493	170.3 ± 0.840	3.2 ± 0.026	4.7 ± 0.030
YGM-MSCA3-3CF	110.2 ± 0.695	167.7 ± 0.817	3.2 ± 0.051	4.6 ± 0.018
PI2542-GF	85.6 ± 0.548	127.6 ± 0.743	2.2 ± 0.027	3.3 ± 0.094
PI2542-KH550-GF	86.3 ± 0.120	131.8 ± 0.480	2.2 ± 0.055	3.4 ± 0.058
PI2542-MSCA1-GF	86.5 ± 0.012	134.3 ± 1.075	2.2 ± 0.094	3.5 ± 0.036
PI2542-MSCA2-GF	90.2 ± 0.320	143.9 ± 1.046	2.3 ± 0.093	3.6 ± 0.072
PI2542-MSCA3-GF	87.0 ± 0.808	137.8 ± 0.486	2.2 ± 0.063	3.5 ± 0.048
PI2542-3GF	90.7 ± 0.876	144.0 ± 0.886	2.3 ± 0.075	3.5 ± 0.051
PI2542-KH550-3GF	93.3 ± 0.753	158.3 ± 1.123	2.4 ± 0.055	3.6 ± 0.089
PI2542-MSCA1-3GF	104.1 ± 0.862	166.5 ± 0.882	2.7 ± 0.017	4.0 ± 0.071
PI2542-MSCA2-3GF	105.9 ± 0.731	168.4 ± 1.140	2.8 ± 0.064	4.3 ± 0.097
PI2542-MSCA3-3GF	104.6 ± 0.788	165.1 ± 0.938	2.7 ± 0.073	4.2 ± 0.040
PI2542-CF	105.7 ± 1.656	161.6 ± 0.875	2.8 ± 0.086	4.3 ± 0.074
PI2542-KH550-CF	112.1 ± 1.771	169.1 ± 0.748	3.0 ± 0.094	4.5 ± 0.026
PI2542-MSCA1-CF	115.3 ± 1.929	171.9 ± 0.712	3.1 ± 0.05	4.6 ± 0.033
PI2542-MSCA2-CF	132.5 ± 1.691	179.0 ± 1.199	3.5 ± 0.045	5.3 ± 0.078
PI2542-MSCA3-CF	120.5 ± 1.751	176.2 ± 1.167	3.2 ± 0.018	4.8 ± 0.064
PI2542-3CF	115.3 ± 0.897	168.9 ± 1.098	3.4 ± 0.063	5.2 ± 0.093
PI2542-KH550-3CF	120.2 ± 0.955	175.6 ± 0.688	3.6 ± 0.055	5.4 ± 0.046
PI2542-MSCA1-3CF	132.0 ± 1.061	181.7 ± 0.963	3.9 ± 0.023	5.9 ± 0.021
PI2542-MSCA2-3CF	136.3 ± 1.173	188.8 ± 0.855	4.0 ± 0.046	6.1 ± 0.086
PI2542-MSCA3-3CF	133.5 ± 1.242	183.3 ± 1.184	3.8 ± 0.095	5.9 ± 0.056

**Table 6:** T5, T10, RM%, and T_g_ of polyimide composites treated with different coupling agents.

Sample	T_5_ (°C)	T_10_ (°C)	RM%	T_g_ (°C)
PI2542-GF	507.6	525.0	56.1	254.7
PI2542-KH550-GF	519.2	536.2	60.5	255.6
PI2542-MSCA1-GF	523.2	539.0	60.7	256.2
PI2542-MSCA2-GF	522.2	539.0	61.0	257.8
PI2542-MSCA3-GF	524.0	540.2	61.7	256.1
PI2542-3GF	496.8	516.6	60.1	254.8
PI2542-KH550-3GF	521.6	538.6	60.6	255.7
PI2542-MSCA1-3GF	524.6	540.8	60.7	256.8
PI2542-MSCA2-3GF	500.8	518.6	61.2	257.6
PI2542-MSCA3-3GF	529.6	543.4	62.8	257.2
PI2542-CF	510.0	527.8	60.2	255.9
PI2542-KH550-CF	518.8	536.2	60.6	256.3
PI2542-MSCA1-CF	523.4	540.2	60.8	257.9
PI2542-MSCA2-CF	501.6	521.4	65.8	258.5
PI2542-MSCA3-CF	514.4	532.2	62.9	257.8
PI2542-3CF	499.0	516.6	61.4	256.2
PI2542-KH550-3CF	499.8	517.8	60.7	256.9
PI2542-MSCA1-3CF	525.4	540.6	60.9	258.5
PI2542-MSCA2-3CF	509.4	528.2	61.7	260.0
PI2542-MSCA3-3CF	522.8	540.2	60.3	258.4

## Data Availability

The original contributions presented in this study are included in the article/Supplementary Materials. Further inquiries can be directed to the corresponding author.

## Supplementary Materials

The following supporting information can be downloaded at: https://www.mdpi.com/article/10.3390/ma19163435/s1, Figure S1: XRD patterns of PI composite carbon fiber and glass fiber.
