# Supplementary material for: Preparation and Application of Macromolecular Silane Coupling Agent for Polyimide-Based Composites
Source: Materials (Basel). 2026 Aug 13;19(16):3435. doi: 10.3390/ma19163435 (PMC13514352; doi:10.3390/ma19163435)
Supplement: Supplementary file 1 [file materials-19-03435-s001.zip › materials-4230115-supplementary.pdf]

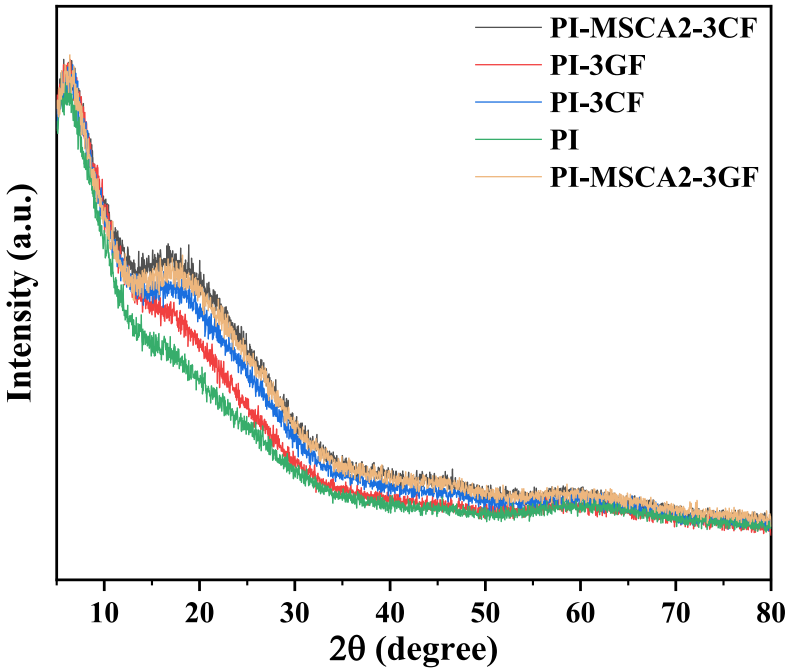

Figure S1: XRD patterns of PI composite carbon fiber and glass fiber.

The X-ray diffraction (XRD) patterns further verified the amorphous network structure of the polyimide (PI) matrix and its composites. All samples exhibited a typical broad amorphous halo in the 2θ range of approximately 15° to 25°, without any sharp crystalline diffraction peaks. This indicated that the asymmetric structures of the PI monomers disrupted the regular packing of the polymer chains, maintaining the macroscopic amorphous nature of the system. Furthermore, the incorporation of fibers and the introduction of the macromolecular silane coupling agent (MSCA2) did not induce crystallization of the matrix, demonstrating the excellent phase stability of the composites. Notably, the diffraction intensity of the amorphous halo for the MSCA2-modified composites (e.g., PI-MSCA2-3CF) showed observable variations compared to the unmodified systems. This alteration in microscopic scattering intensity indirectly corroborated the "physical entanglement" effect at the interface. Because MSCA2 possessed a rigid backbone highly similar to the PI matrix, its long macromolecular chains deeply diffused and entangled with the matrix at the interfacial region. Such strong interactions restricted the mobility of local polymer segments and altered the short-range ordering and packing density at the interface. Consequently, the XRD results not only confirmed the structural stability of the amorphous phase but also provided microstructural evidence for the interfacial reinforcement mechanism driven by long-chain physical entanglement.
